# Supplementary material for: Organometallic Pillarplexes That Bind DNA 4-Way Holliday Junctions and Forks
Source: J Am Chem Soc. 2023 Jun 15;145(25):13570–80. doi: 10.1021/jacs.3c00118 (PMC10311459; doi:10.1021/jacs.3c00118)
Supplement: Supplementary file 1 — ja3c00118_si_001.pdf [file ja3c00118_si_001.pdf]

Supplementary information

Organometallic Pillarplexes that bind DNA 4-way Holliday Junctions and forks

James S. Craig,[a] Larry Melidis,[a] Hugo D. Williams,[a] Samuel J. Dettmer,[b] Alexandra A. Heidecker,[d,e] Philipp J. Altmann,[d,e] Shengyang Guan,[d,e] Callum Campbell,[b] Douglas F. Browning,[c] Roland K.O. Sigel,[f] Silke Johannsen,[f] Ross T. Egan,[b] Brech Aikman,[d] Angela Casini,[d] Alexander Pöthig,[d,e,*] Michael J. Hannon[a,b,*]

a Physical Sciences for Health Centre, b School of Chemistry, c School of Biosciences, University of Birmingham, Edgbaston, Birmingham B15 2TT, UK. Email: m.j.hannon@bham.ac.uk
d Department of Chemistry, e Catalysis Research Centre, Technical University of Munich (TUM), Lichtenbergstr. 4, 85748 Garching b. München, Germany. Email: Email: alexander.poethig@tum.de
f Department of Chemistry, University of Zürich, Winterthurerstr. 190, 8057 Zürich, Switzerland.

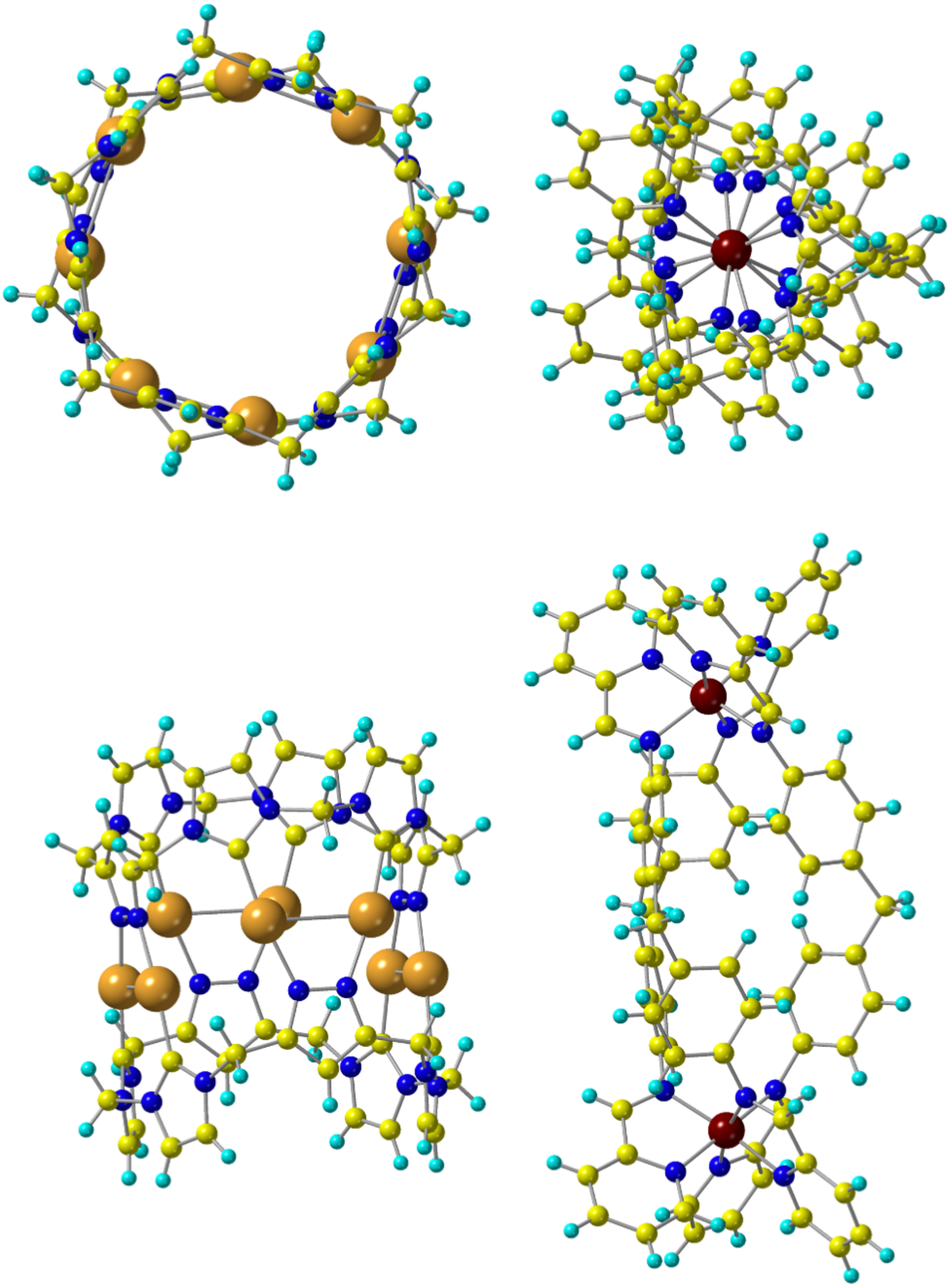

Figure S1a Comparative views of the pillarplex (left) and cylinder (right) from the top and side.

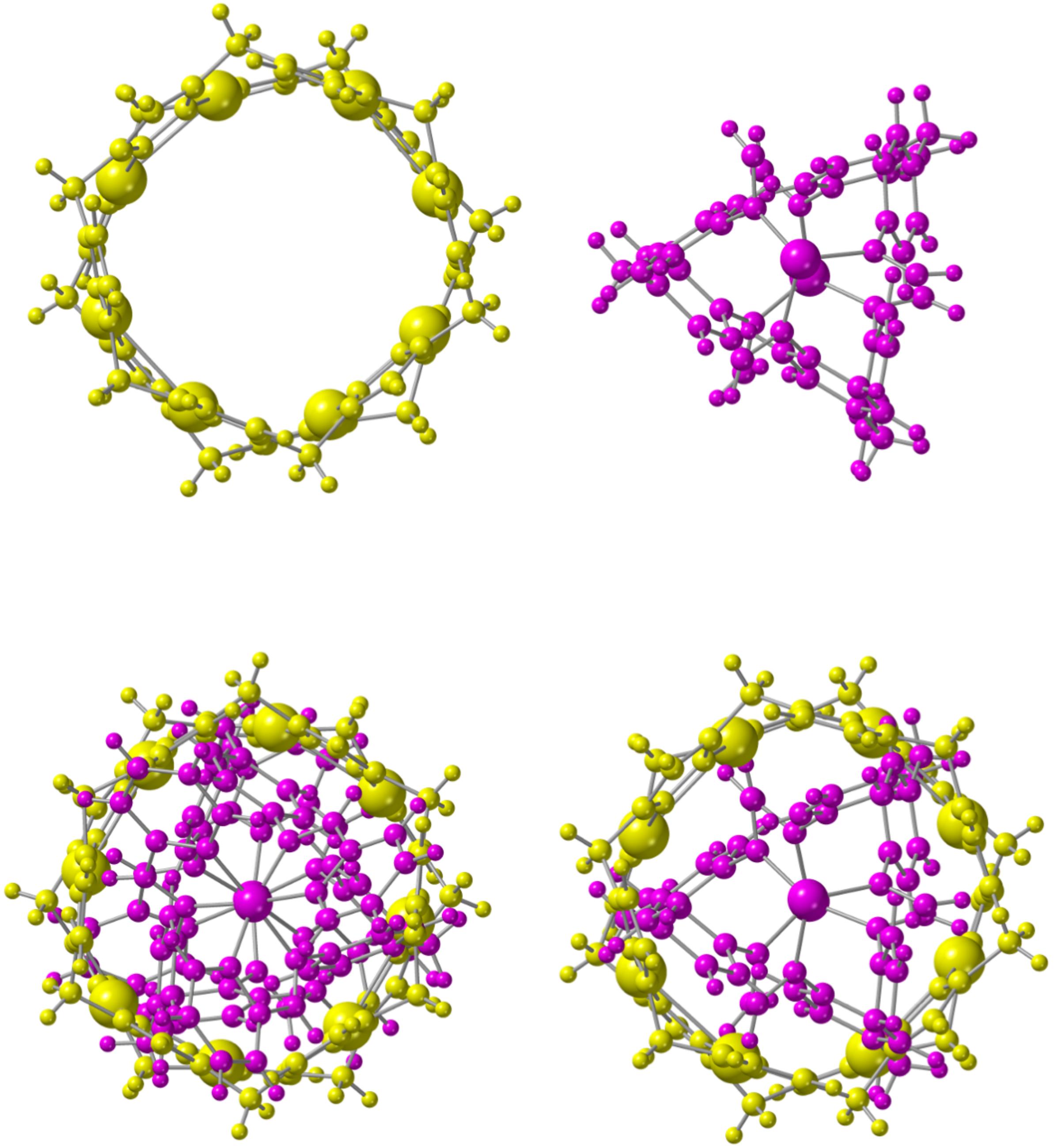

Figure S1b Comparative views of the pillarplex (top left) and the central core of the cylinder which binds the 3WJ cavity ie with the pyridyls cut away (top right). Overlay of the full cylinder structure with the pillarplex (left) and just the central core of the cylinder with the pillarplex (right).

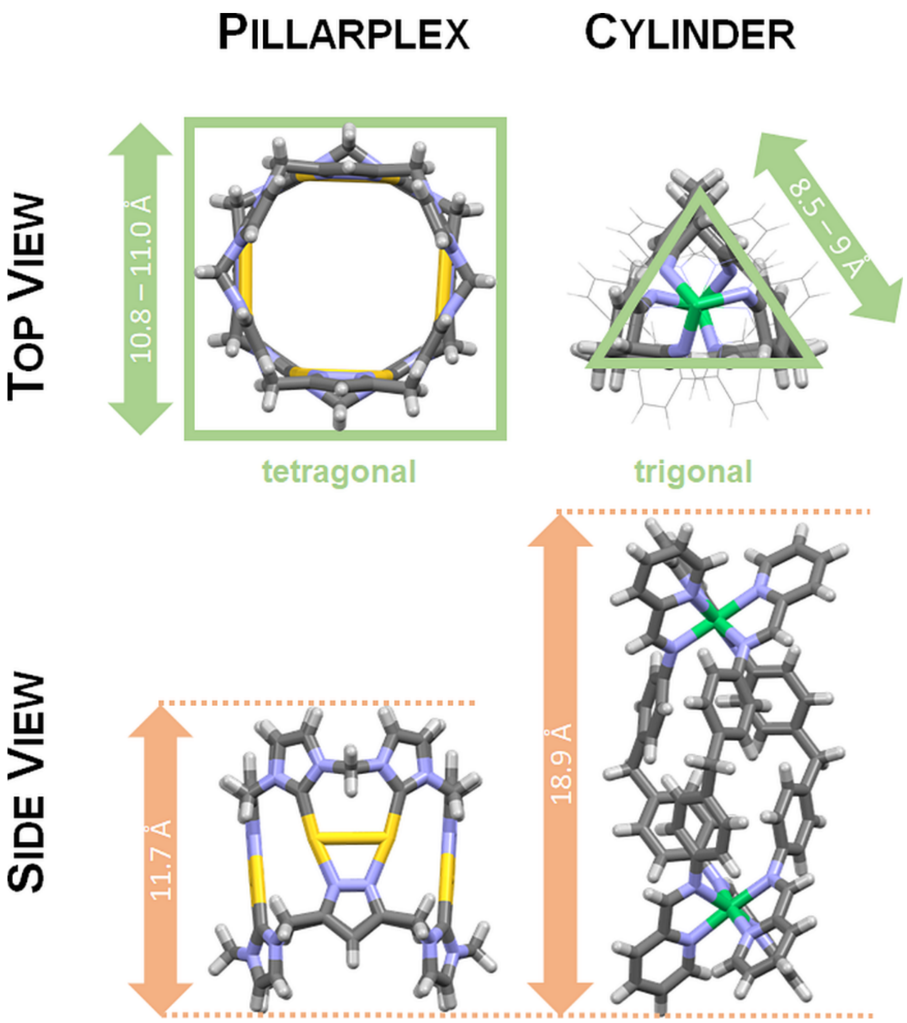

Figure S1c: Top: Comparative views of the pillarplex (left) showing the tetragonal arrangement aromatic surfaces along the coinage-metal pairs, and the trigonal central core of the cylinder which binds the 3WJ cavity (with the pyridyls shown as wireframe) (right). Note: Distances have been calculated from centroids between the furthest distanced CH2-groups: pillarplex H4A/B & H4A'/B' and H25A/B & H26A/B (CSD code: HAKQAO); Cylinder H10/11 & H10A/11A & H21/21A (CSD code: NITBIB). Bottom: Comparative side views of the pillarplex (left) and the the cylinder showing the significant difference in height. Note: Height distance was taken between centroids calculated from the distal hydrogen atoms (pillarplex: H3 H3' H7 H7' & H15 H15' H23 H23', cylinder: H2 H13 H23 & H2A H13A H23A.

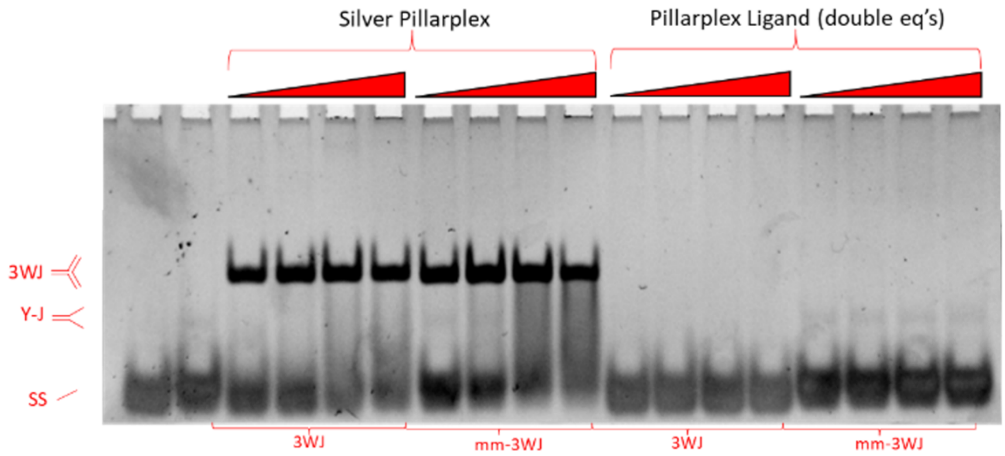

Figure S2: Shows a PAGE gel of 3WJ and mm-3WJ DNA strands incubated with Ag pillarplex and pillarplex ligand at varying ratios. The gel is analogous to main paper Figure 3 (Au pillarplex and Ni cylinder). Gel lanes read from left to right. Controls in lanes 1 (3WJ strands alone) and 2 (mm-3WJ strands alone). 3WJ is mixed with Ag pillarplex or ligand at 0.5,1, 2 and 4 eq (lanes 3-6 and 11-14), and mm-3WJ mixed with Ag pillarplex or ligand at 0.5,1,2,4 eq (lanes 7-10 and 15-18).

The Ag pillarplex stabilises the 3WJ and mm-3WJ structures. In both cases a shifted 3WJ band is observed which is very similar to that observed with Au pillarplex. At high loading some smearing below the 3WJ is observed, however (in contrast to Au pillarplex) we do not see a pronounced Y-shaped band at higher loading. The Ag pillarplex has poorer solution stability than the Au pillarplex, and there is potential for release of silver cations (from pillarplexes not bound in the 3WJ cavity) which may then bind to the DNA.

The free polyaryl ligand from which the pillarplexes are constructed shows no binding to DNA.

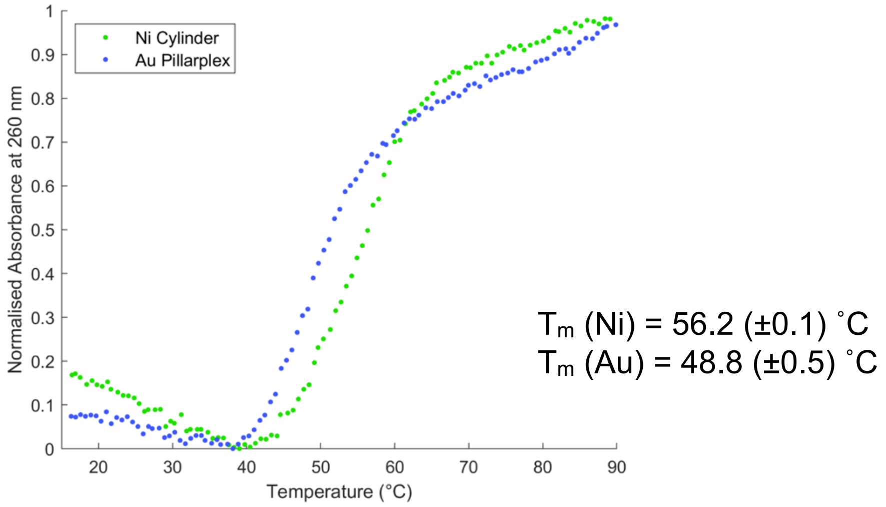

Figure S3a: Representative data for the 3WJ UV-Vis melting experiments (14 bases per strand) with Ni cylinder (green) and with Au pillarplex (blue).

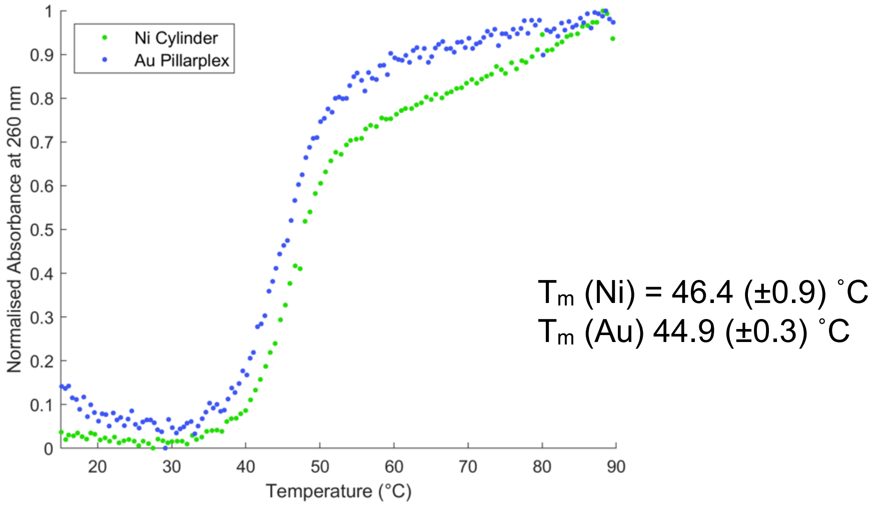

Figure S3b: Representative data for the mm3WJ UV-Vis melting experiments (14 bases per strand) with Ni cylinder (green) and with Au pillarplex (blue).

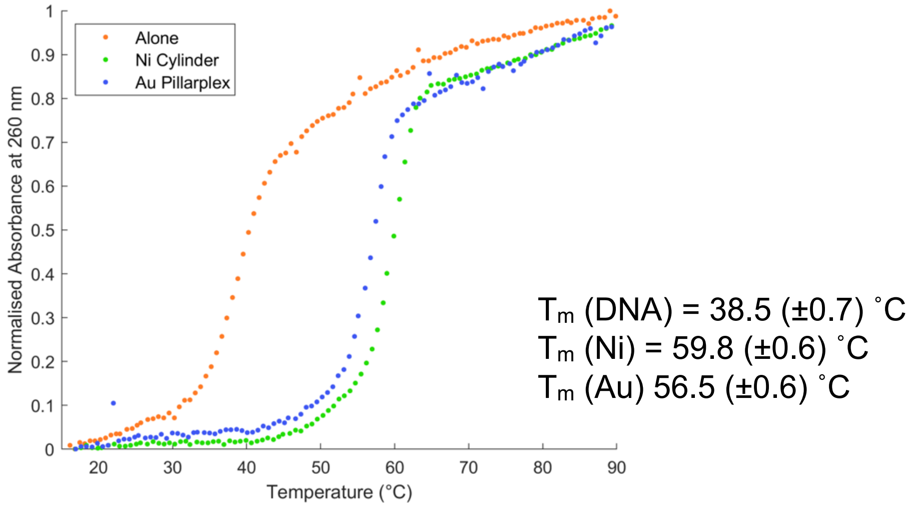

Figure S4: Representative data for 3WJ18 UV-Vis melting experiments (18 bases per strand) alone (red) with Ni cylinder (yellow) and with Au pillarplex (blue).

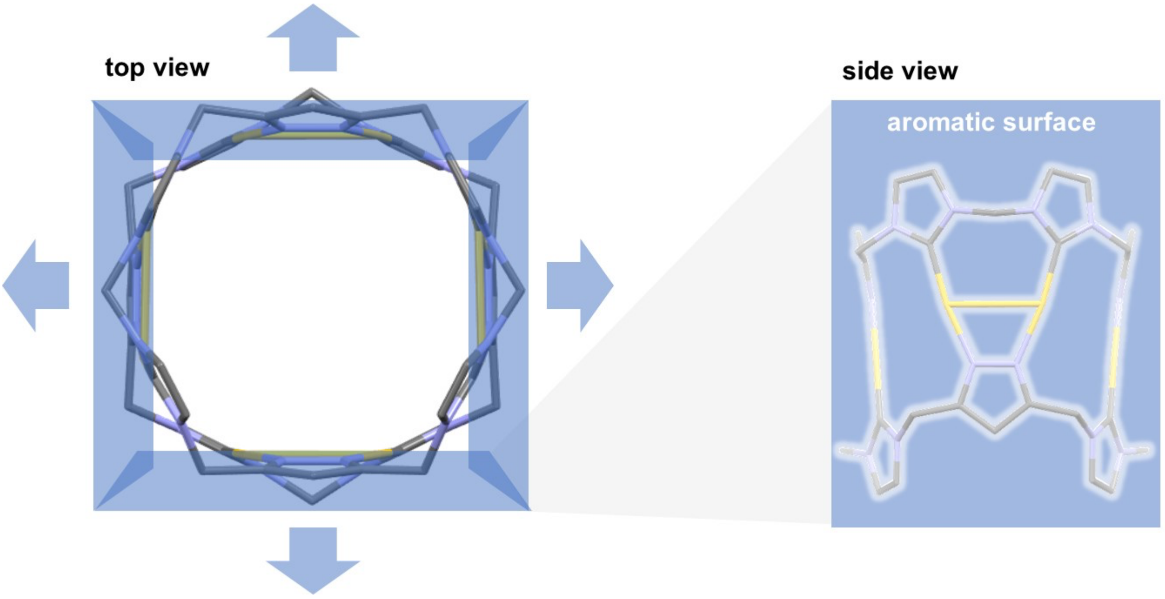

Figure S5 Views of the pillarplex emphasising the tetragonal arrangement of surfaces within the structure

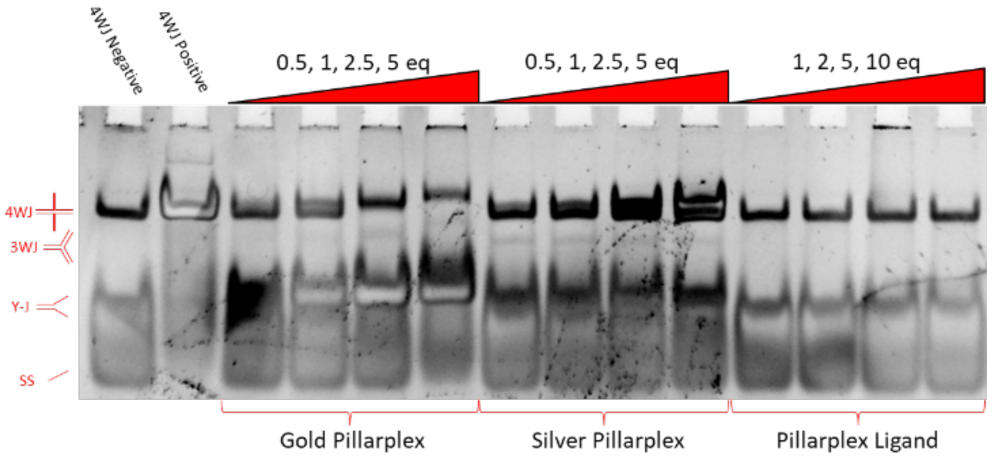

Figure S6 Shows a PAGE gel, of a 4WJ incubated with Au and Ag pillarplexes and the free pillarplex ligand at varying ratios. Lanes 1-6 (left to right) are also shown in the main paper Figure 5. S1,S2,S3 and S4 are present in all lanes, with lanes 1 and 2 representing controls (Lane 1 - strands alone. Lane 2 - in presence of cations 2mM Mg^{2+} and 450mM Na^{+}). Complexes or ligand are added in at 0.5,1,2.5 and 5 eq. ratios (compound: 4WJ). Lanes 2-6 Au pillarplex, lanes 7-10 Ag pillarplex and lanes 11-14 pillarplex ligand.

The Ag pillarplex (as the Au pillarplex) binds the 4WJ (for which the band broadens and is slightly shifted), with a faint band also observed for the discrete three-strand structure and a larger band for the Y-fork duplex DNA. It is interesting that the 4WJ band experiences less shift at high loading than with Au pillarplex, though this may reflect a lower pillarplex concentration given the lower stability of the silver complex. Once again controls with the ligand do not indicate free-ligand binding.

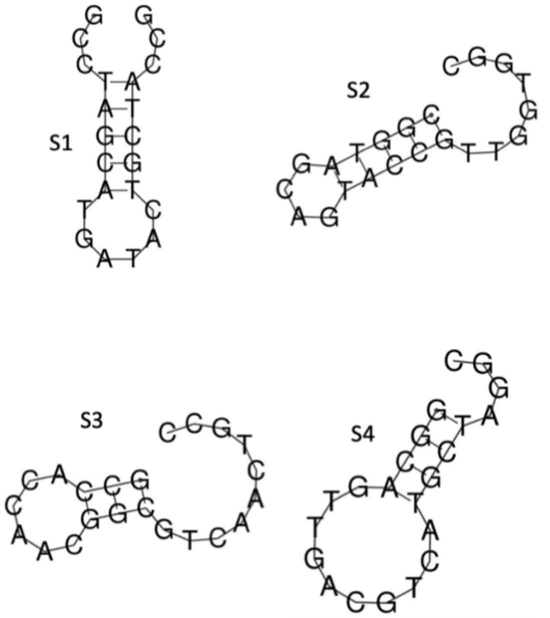

Figure S7: Example of how the individual single strand oligos from the 4WJ might fold. Produced using the nucleic acid Fold structure prediction software from the Matthews Lab at U. Rochester available at:
https://rna.urmc.rochester.edu/RNAstructureWeb/Servers/Predict1/Predict1.html
Although the four oligos are the same length, some small differences in mobility are observed in the gel in main paper Figure 6 likely indicating different levels of folding.

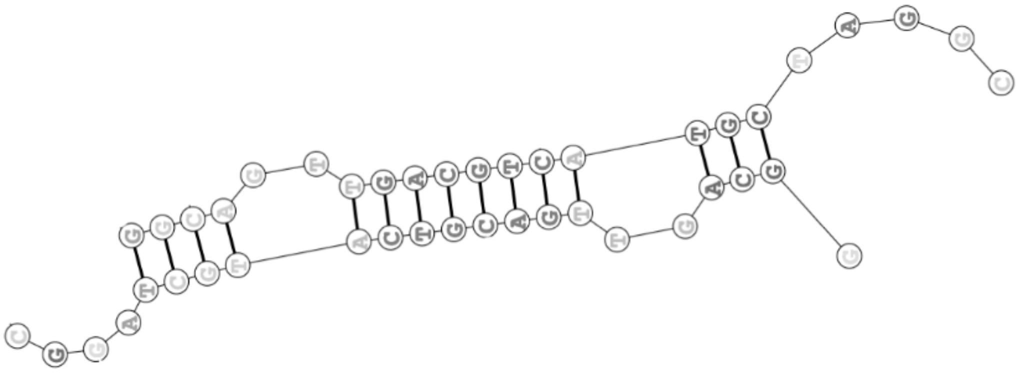

Figure S8: Example of how two S4 oligos might interact to form a dimer. The gel in main paper Figure 6 indicates that S4 produces dimer in absence of pillarplex. Produced using the nucleic acid Fold structure prediction software from the Matthews Lab at U. Rochester available at:
https://rna.urmc.rochester.edu/RNAstructureWeb/Servers/Predict1/Predict1.html
The prediction used a sequence of two S4 strands connected by an XXXXX spacer which was then removed from this graphical representation.

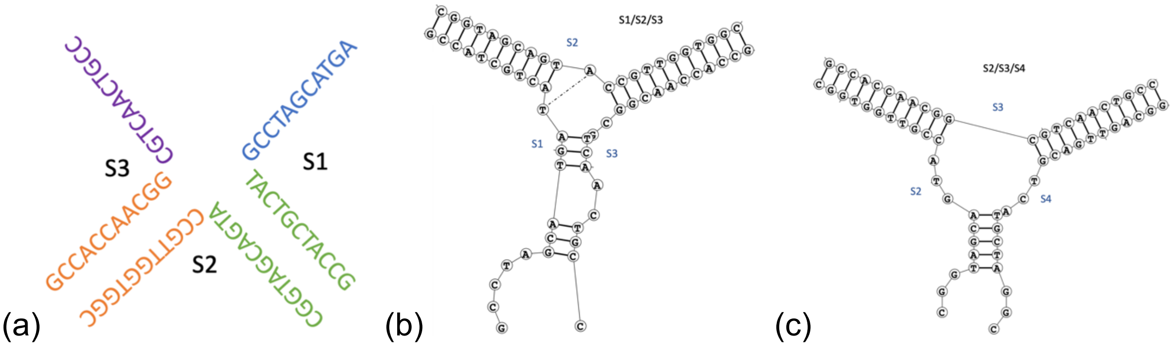

Figure S9: Examples of how three oligo sequences might interact to form a 3WJ. The 3-strand bands observed with cylinder may represent formation of an open forked version of the 4WJ (S9a) but more likely the ss arms of that structure will interact (S9b) in this example potentially leading to a 3WJ with a 2-base bulge. This is especially true when cylinder is present to bind in the cavity and stabilise the 3WJ form. Previous work has confirmed the ability of cylinders to bind to 3WJ containing bulges at the junction point as well as perfect 3WJs (Malina, J.; Hannon, M.J.; Brabec, V. Recognition of DNA Three-Way Junctions by Metallosupramolecular Cylinders: Gel Electrophoresis Studies. *Chem. Eur. J.* **2007**, *14*, 3871-3877) Structures produced using the nucleic acid Fold structure prediction software from the Matthews Lab at U. Rochester available at:
https://rna.urmc.rochester.edu/RNAstructureWeb/Servers/Predict1/Predict1.html
The prediction used the sequence of the three strands connected by XXXXX spacers and combined into a single strand, with the XXXXX spacers removed from this graphical representation.

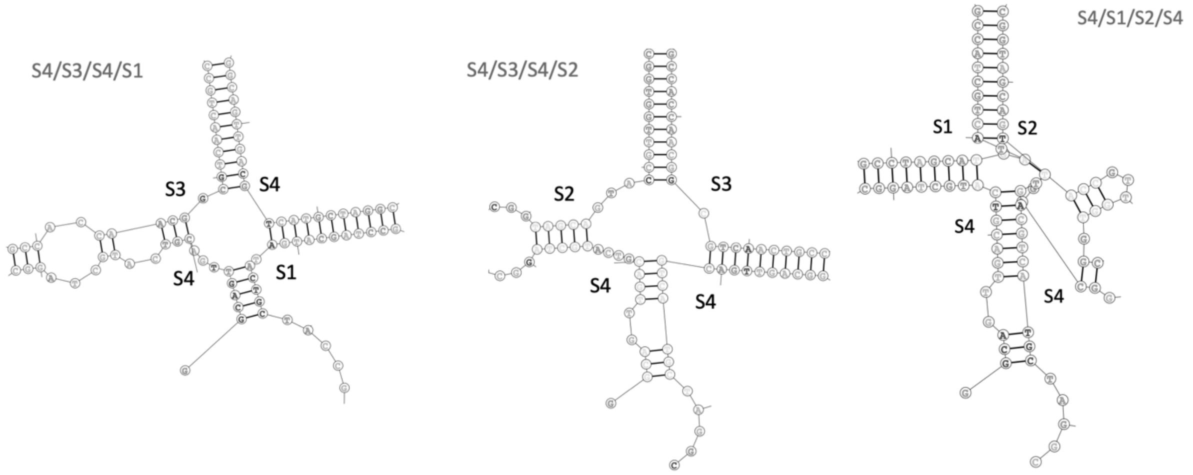

Figure S10: Example of how three oligo sequences might interact to form a tetramer. The gel in main paper Figure 6 indicates that combinations including S4 can produce small amounts of a tetramer in absence of the fourth sequence strand, with this seen very weakly in absence of pillarplex, and more strongly in presence of pillarplex or cylinder. Produced using the nucleic acid Fold structure prediction software from the Matthews Lab at U. Rochester available at:
https://rna.urmc.rochester.edu/RNAstructureWeb/Servers/Predict1/Predict1.html
The prediction used a sequence of two S4 strands and two others with each sequence connected together into a single strand by an XXXXX spacer which was then removed from this graphical representation.

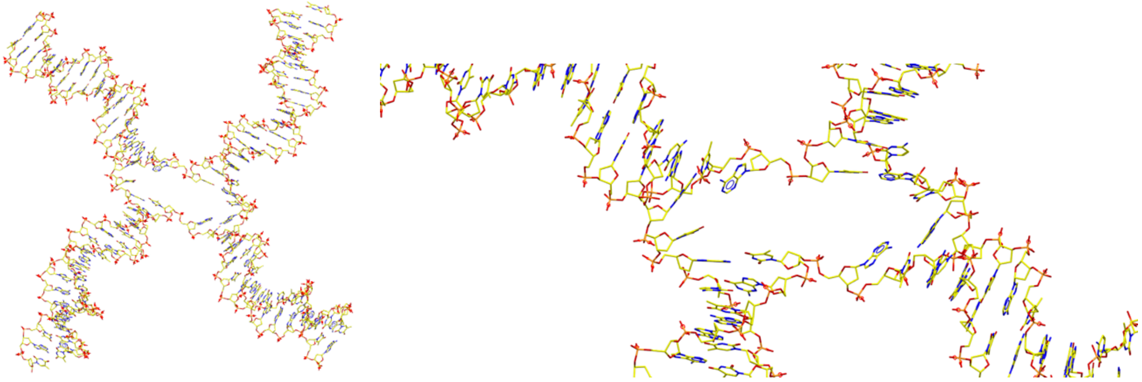

Figure S11a: Starting position of the DNA 4WJ as extracted from pdb 1XNS (crystal structure) and used in simulations, illustrating the partially open cavity. The second image is a close-up (slightly rotated) to better illustrate the partially open cavity.

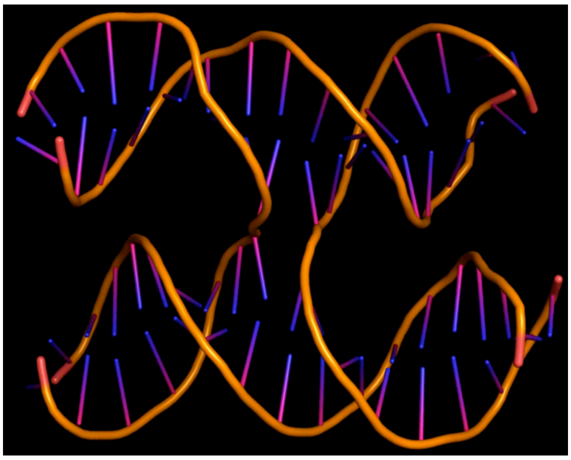

Figure S11b: Example of the closing of the DNA 4WJ into the closed form in simulations of the free DNA, in absence of pillarplex.

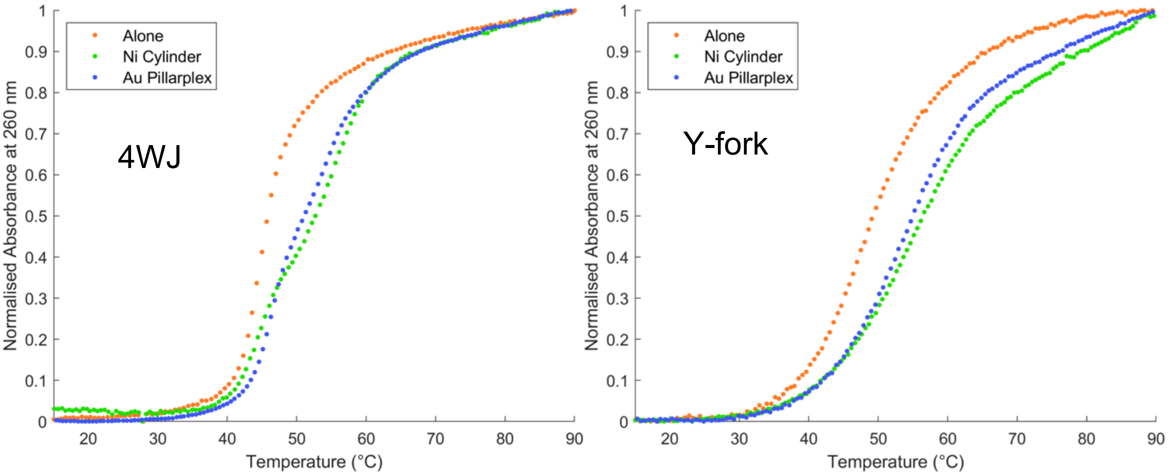

T_m (DNA) = 45.0 (±0.1) ˚C
T_m (Ni) = 45.1 (±0.1); 55.1 (±0.1) ˚C
T_m (Au) = 46.2 (±0.1); 53.2 (±0.6) ˚C

T_m (DNA) = 47.1 (±0.1) ˚C
T_m (Ni) = 55.1 (±0.1) ˚C
T_m (Au) = 55.0 (±0.7) ˚C

Figure S12: Representative UV-Vis melting experiments data for the 4WJ (left) and a Y-shaped fork formed from S2, S3 (right) with Ni cylinder (green) and with Au pillarplex (blue). Biphasic melting behaviour is apparent with 4WJ and is more striking for the Ni cylinder and suggesting a multi-step melting process. Binding to both 4WJ and Y-forks is apparent, which is consistent with electrophoresis studies. The similarities in T_m values between 4WJ and fork, may suggest the involvement of fork in the 4WJ melting process.

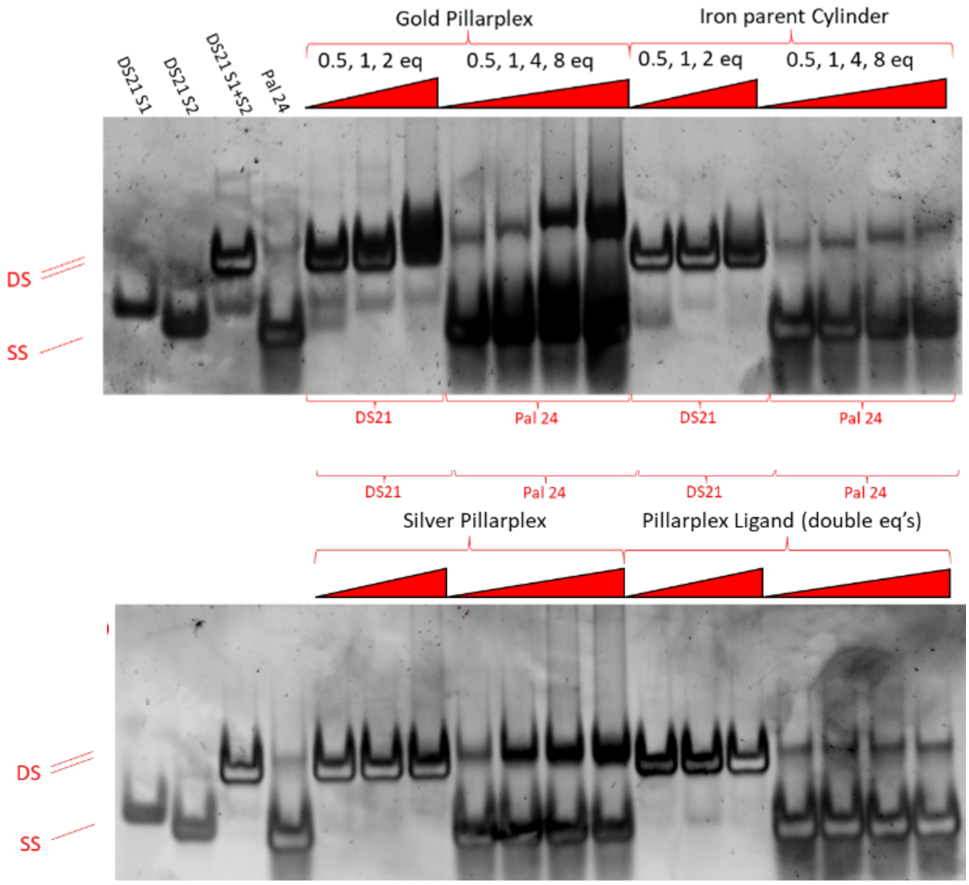

Figure S13. PAGE gels of DS21 and Pal24 incubated with gold Pillarplex, iron Cylinder, silver Pillarplex and Pillarplex ligand at varying ratios. Controls in lanes 1-4, then DS21 mixed with a complex at 0.5,1 and 2 eq (lanes 5-7 and 12-14), and Pal24 mixed with a complex at 0.5,1,2,4 eq (lanes 8-11 and 15-18). The silver pillarplex promotes the formation of duplex DNA for Pal24 but its effects are less striking than those of the Au pillarplex. The pillarplex ligand shows no evidence of binding to the DNA.

.

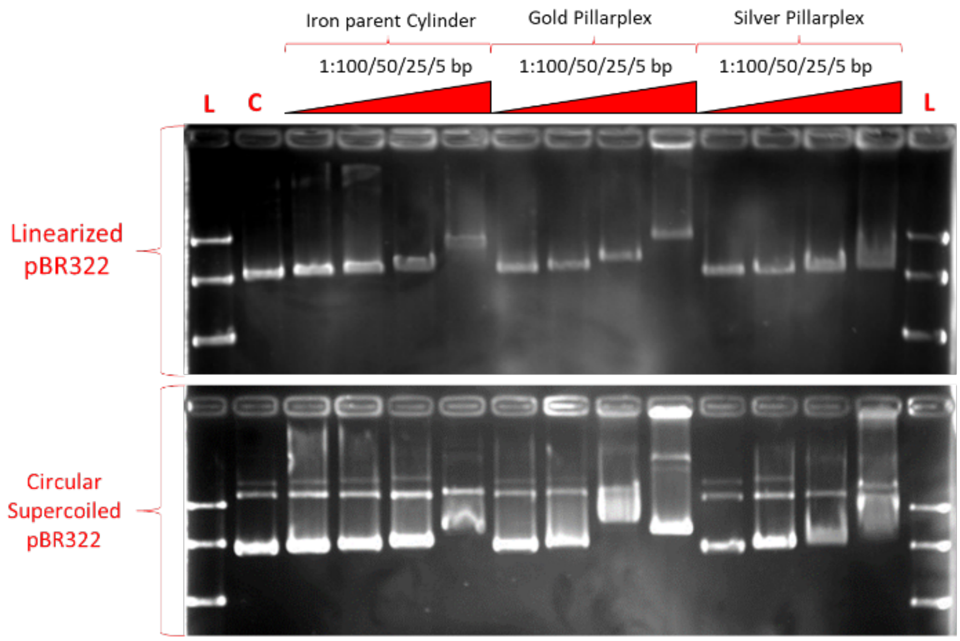

Figure S14. Agarose Gel Electrophoresis Studies of Circular supercoiled (bottom) and linearized (top) pBR-322 plasmid DNA with varying ratios of Fe cylinder, Au pillarplex and Ag pillarplex. Lanes 1 and 15 are a DNA Ladder (L), and lane 2 the plasmid DNA alone as control. The Ag pillarplex shows similar behavior to the gold pillarplex but at higher loading values, consistent with some degradation of Ag pillarplex in the experiment leading to lower values of pillarplex in solution.
(For further information on the relationship of circular plasmid gel shifts to unwinding see for example: The unwinding of circular DNA by intercalating agents as determined by gel electrophoresis. Dougherty, G. *Biosci Rep.* **1983**, 3(5), 453-60. doi: 10.1007/BF01121956).

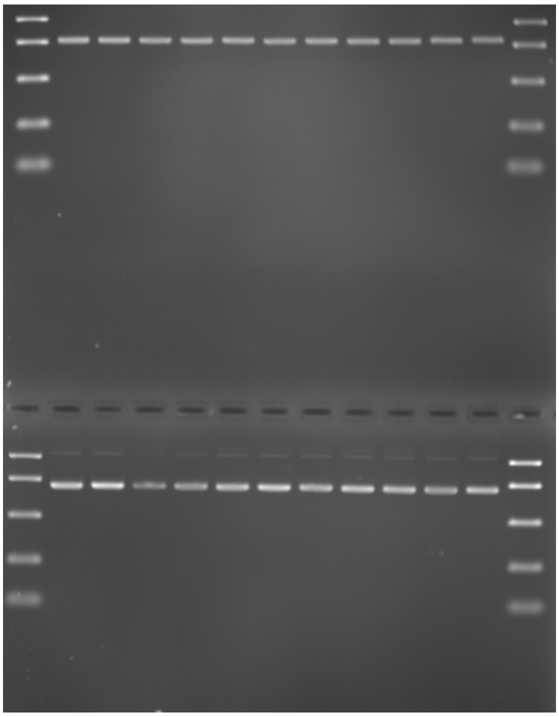

Figure S15: Agarose gel electrophoresis gel of pillarplex ligand with PBR-322 linearized plasmid DNA (top) and circular plasmid DNA (bottom). 30:1, 20:1, 15:1, 10:1, 7.5:1, 5:1, 4:1, 3:1, 2:1, 1:1 DNA base pairs to ligand. Ratios are 2x the concentration of pillarplexes in Figure S14, to account for the presence of 2 ligands in each Pillarplex.   No evidence of binding is observed.

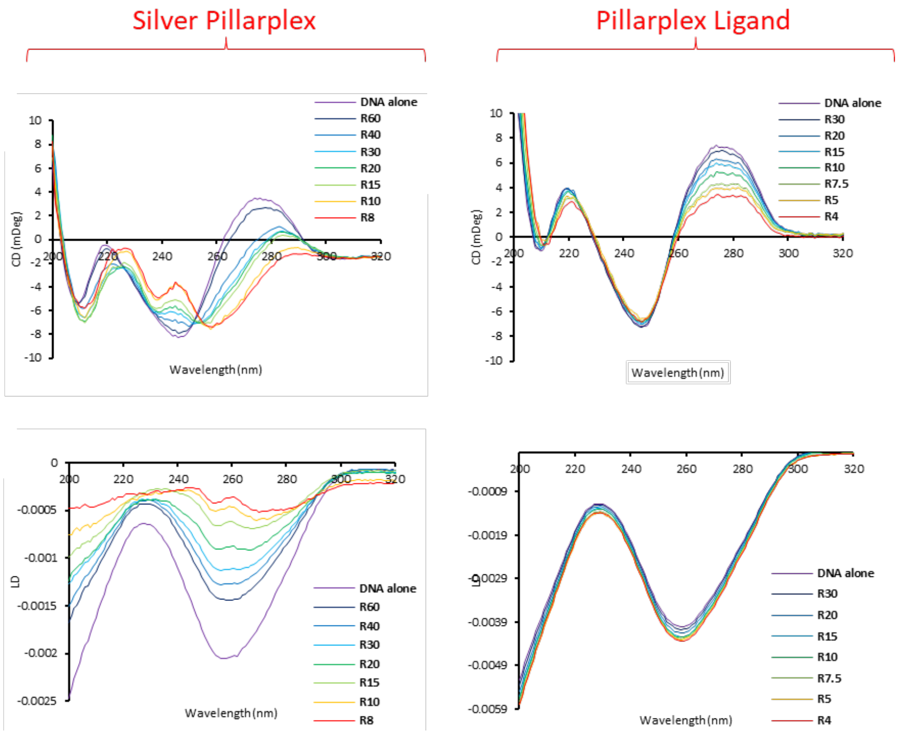

Figure S16. Circular dichroism (top) and flow Linear Dichroism (bottom) spectroscopic studies of the of Ag pillarplex and pillarplex ligand with CT-DNA. The R value is the ratio of DNA base pairs to complex. Spectra are acquired by titration of a solution (containing complex of interest) and 2x DNA/buffer solution to an initial solution of 100 μM CT-DNA (bp) with spectra being recorded after each titration addition. The CD and LD spectra of the Ag pillarplex bear similarities to those of the Au pillarplex, though the effects are less striking. In particular in the LD, for Ag pillarplex, the signals do not go positive within this concentration range though if the Ag pillarplex is degraded in the solution the real concentrations may be lower.

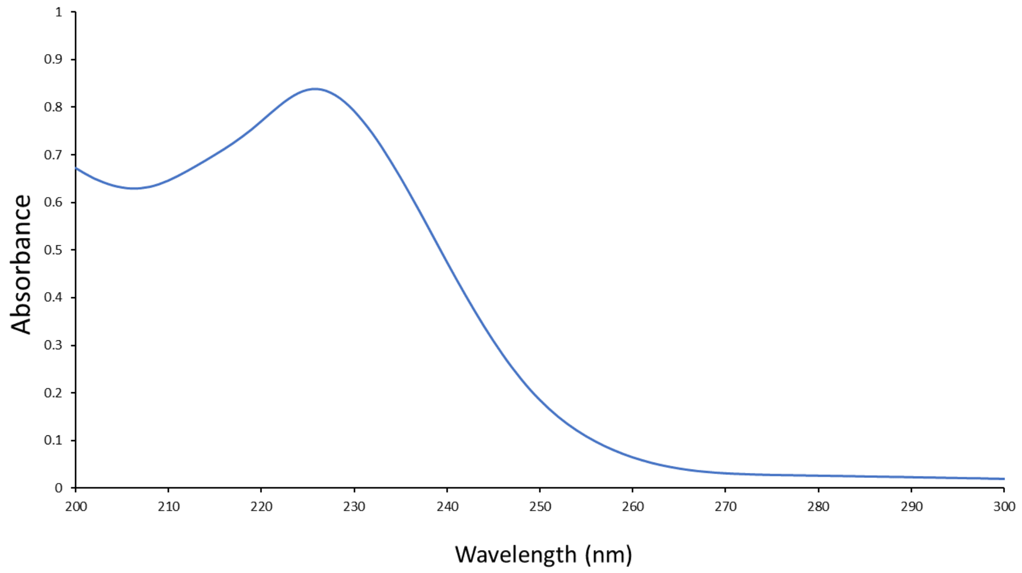

Figure S17 Ag pillarplex absorbance spectrum at 10 μM concentration in water.

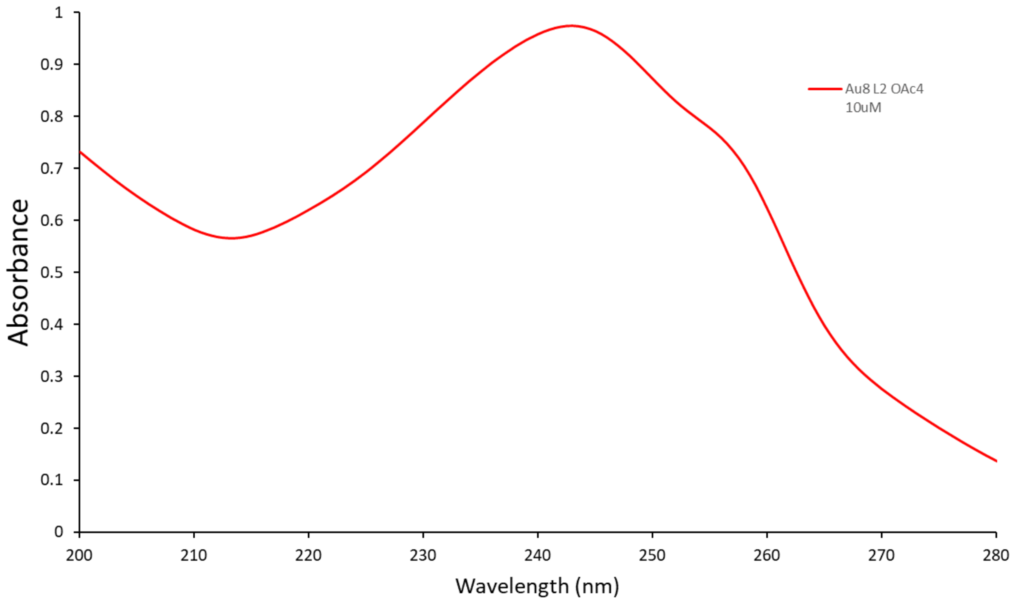

Figure S18: Au pillarplex absorbance spectrum at 10 μM concentration in water.

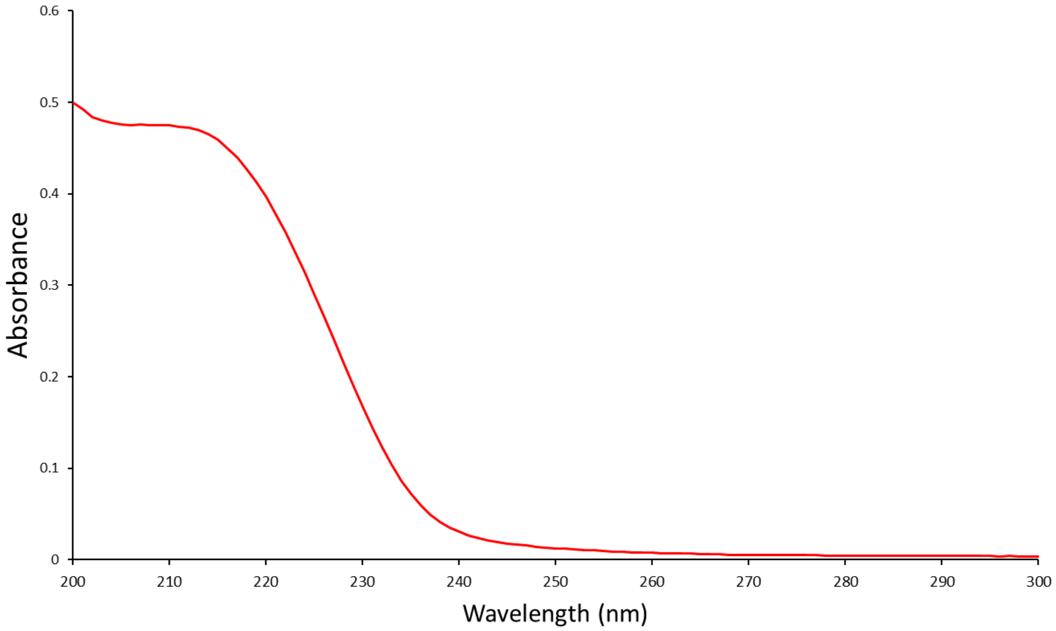

Figure S19a: Pillarplex ligand absorbance spectrum at 10 μM concentration in water.

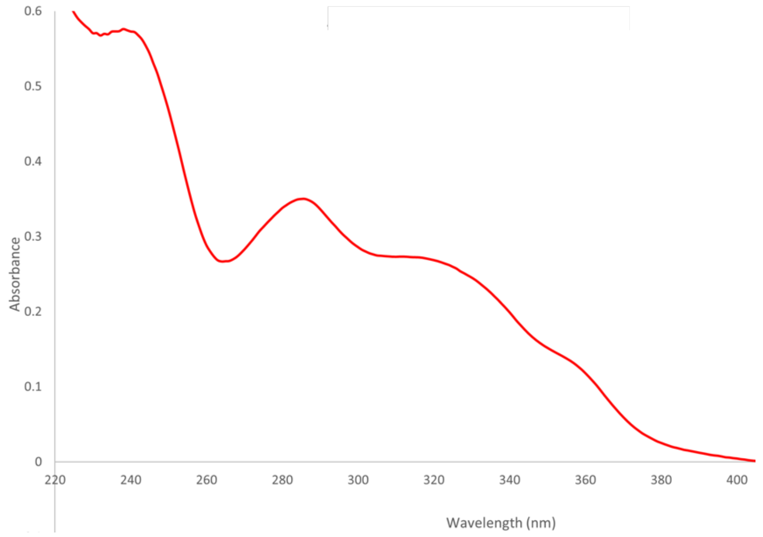

Figure S19b: Nickel(II) cylinder absorbance spectrum at 10 μM concentration in water.

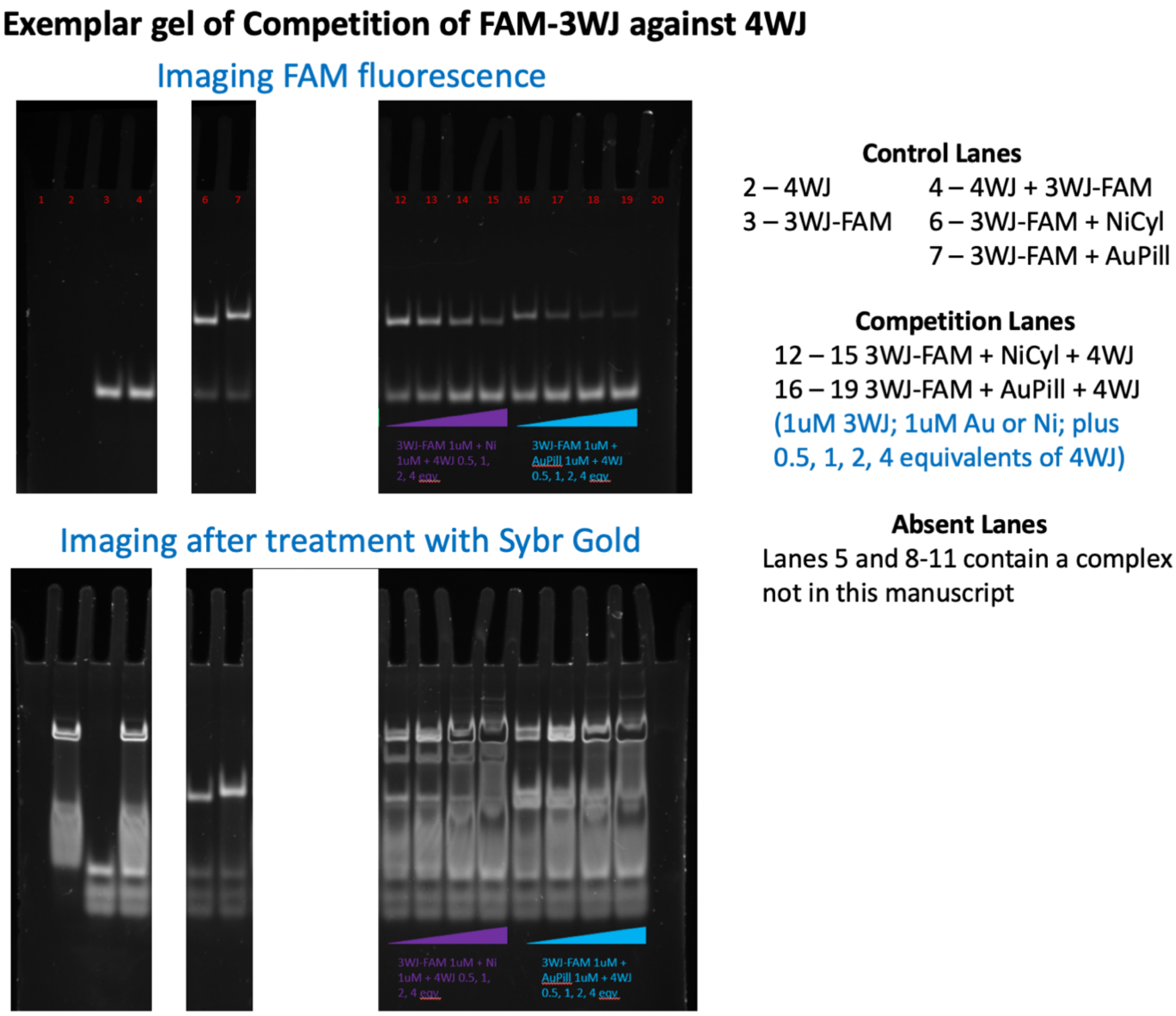

Figure S20: An exemplar gel of the competition of FAM-3WJ against 4WJ with FAM imaging and then subsequent Sybr Gold staining and re-imaging. The gel demonstrates that in the case of the Ni cylinder it is both a 3-strand complex of the 4WJ and the 4WJ which compete against the FAM-3WJ which is consistent with the observations in main manuscript Figs 5 and 6.

Table ST1: Cell cytotoxicity results

	Mean IC_{50} ± SD (μM)			
	A549<br>Human epithelial lung carcinoma		SKOV-3<br>Human ovarian cancer	
	24h	*72h*	*24h*	*72h*
Au pillarplex	41.3 ± 4.1	11.3 ± 1.8	>50	12.4 ± 1.9
Ag pillarplex	5.7 ± 2.0	6.95 ± 2.85	9.7 ± 2.1	5.45 ± 0.75
cisplatin	>100	11.8 ± 1.5	>100	15.4 ± 2.2

Experimental Details

Biophysics experimental: Milli-Q water (18.2 MΩ) was used throughout all biophysical work. For Circular and Linear dichroism, DNA samples were made up from Calf thymus DNA sodium salt (Sigma Aldrich) by dissolving in milli-Q water(18.2 MΩ) and washed using a 10 kDa MWCO centrifuge tube (Sartorius, Vivaspin, 10 ml). The solution was then quantified by UV-Vis spectroscopy (Cary 5000 NIR spectrometer) by ε (260 nm) = 13,200 mol^{-1} dm^3 cm^{-1} to give a concentration in DNA base pairs. This stock solution was kept frozen with fresh aliquots being taken out for each experiment. Fresh buffer was made up before each experiment (see experimental for specifics). Complexes were dissolved in Milli-Q water (18.2 MΩ) only with fresh solutions being used for each batch of experiments.

Circular dichroism (CD): spectra were recorded on a Chirascan+ spectrometer (Applied Photophysics limited). The samples were scanned in a 1 cm path length cuvette between 800 and 200 nm with 3 repeats at 1 nm step size and 0.5s dwell time per point. Titrations were carried out at a constant concentration of CT-DNA, sodium chloride (10 mM) and sodium cacodylate buffer (1 mM, pH 7.3) by adding compensating solutions of 2x DNA/Buffer of equal volume to the titre of the complex solution. The concentration of complex in the cuvette was increased step wise by adding set volumes of a stock complex solution. The R value refers to the ratio of DNA base pairs to complex, i.e. R60 = 60bp for every 1 complex, R4 = 4bp per complex. CT-DNA concentration was 100 μM in DNA base pairs.

Flow Linear Dichroism (LD): was carried out on the same Chirascan+ spectrometer (Applied Photophysics limited) using the LD accessory (Applied Photophysics limited). The LD cell has an angular gap of 0.25 mm giving an overall path length of 0.5 mm. Samples volumes began at 150 μl and stopped at 250 μl. The cell was rotated at 40 revolutions per second to optimise the DNA signal. The titration series was carried out the same as in the CD studies, with a 3-minute incubation time at a lower revolution speed of 4 revolutions per second. CT-DNA concentration was 100 μM in DNA base pairs.

Polyacrylamide gel electrophoresis (PAGE): Studies were performed using following oligonucleotide sequences (from Eurofins Germany) which were purified by reverse-phase HPLC.

DNA melting: The stability of the DNA three-way junction (3WJ18 and 3WJ) with different junction binding agents was monitored by measuring the absorbance at 260 nm (bandwidth, 1nm; average time 4 s; heating rate, 1.0 ˚Cmin-1; measurement interval, 0.5 ˚C) with increasing temperature. A 1 cm path length, masked, quartz cuvette, and a peltier-temperature controller were used in a Cary 5000 UV-Vis-NIR spectrophotometer. The 3WJ18 structure was composed of 3 separate oligonucleotides, the solutions measured contained 1 μM of each oligo and 1 μM of metal complex and were made up in Sodium Cacodylate buffer (10 mM, pH 7.4) and NaCl (100 mM). The 3WJ melting experiments were carried out in the same manner but with 2 μM each oligo and 2 μM metal complex. The melting temperature (Tm) was calculated using the thermal heating program's built-in smoothing function and first derivative calculation. Each condition was made in triplicate and the T_m reported is the average of three runs.

DS21:
S1 = 5’ - CCTTCACGCGAACGTAATCCT - 3’
S2 = 5’- AGGATTACGTTCGCGTGAAGG - 3’

PAL24:
S1 = 5’-CTTGAGCTTGAGCTCAAGCTCAAG-3’

mm-3WJ:
S1 = 5’-CGGAACGGCACTCG-3’
S2 = 5’- CGAGTGCTGCGTGG-3’
S3 = 5’-CCACGCTCGTTCCG-3’.

3WJ:
S1 = 5’-CGGAACGGCACTCG-3’
S2 = 5’- CGAGTGCAGCGTGG-3’
S3 = 5’-CCACGCTCGTTCCG-3’

HJ/4WJ:
S1 = 5’-GCCTAGCATGATACTGCTACCG-3’
S2 = 5’-CGGTAGCAGTACCGTTGGTGGC-3’
S3 = 5’-GCCACCAACGGCGTCAACTGCC-3’
S4 = 5’-GGCAGTTGACGTCATGCTAGGC-3’

3WJ18
S1 = GTGGCGAGAGCGACGATC
S2 = GATCGTCGCAGAGTTGAC
S3 = GTCAACTCTTCTCGCCAC

Fluorescent PAGE experiments: PAGE Polyacrylamide gels were prepared by mixing 25 ml of 29:1 acrylamide/bis-acrylamide (National Diagnostic, protogel) with 5 mL of 10x Tris-Boric acid buffer (890 mM each, pH 8.3, or pH 7.05 adjusted with HCl) and 20 ml of Milli-Q water. To this 400 µL of a 10% w/v ammonium persulfate/water solution and 40 µL of TEMED were added to initialise polymerisation. This is then immediately poured between 2 glass plates and a comb inserted at the top this is then left to set for 1 hr. Samples were made up to 10 µl containing 1 µM of each strand 1xTBN buffer (89 mM Tris, 89 mM Boric acid, 10 mM NaCl, either pH 8.3 or 7.05) and the indicated ratio of Complex or competitor. DNA, water, and buffer were mixed in solution before addition of the stated ratios of complex and additional components. Samples were then incubated at 37 degrees Celsius for 1 hr. 5 µl of 30% w/v glycerol was then added to each sample and the sample was then centrifuged, mixed, and pipetted into the wells on the gel. The Gel was run at 140 V for 2 hours in 1xTB buffer. The gel was then removed from the plates and stained using SYBR™ Gold Nucleic Acid Gel Stain (Thermofisher scientifc) in 1xTB buffer for 45 minutes before imaging on a bio-rad ChemiDoc fluorescent imager with 305 nm excitation. DNA structures were at 1 µM in the samples with equal concentrations of each strand needed for their respective structures, complex concentrations are indicated in ratios to this.

3WJ Radiolabelled PAGE: To confirm that fluorescent gel results were not affected by any potential fluorescence quenching by metal complexes, 3WJ gels were also repeated using radiolabelled DNA. The results confirmed that SYBR gold staining was sutiable for studying the DNA binding of the Ni cylinder and Au pillarplex in gels. 1.2 µL of S1 was radiolabelled using 1 µL of adenosine triphosphate γ 32P (Perkin Elmer) at the 5' end using 2 µL of T4 bacteriophage polynucleotide kinase (New England Biolabs) by incubating them at 37 degrees Celsius for 1 hr in 2 µl of 10xPNK buffer (New England Biolabs) made up to 20 µL with nuclease free water (ThermoFisher Scientific). This solution was heated to 80 °C for 3 minutes to inactivate the enzyme and before being purified using a QAIquick nucleotide removal column (Qiagen), this was washed twice after binding to the column, and then eluted with 60 µL of nuclease free water to leave a 4 µM solution of radiolabelled S1*. PAGE Polyacrylamide gels were prepared by mixing 25 ml of 29:1 acrylamide/bis-acrylamide (National Diagnostic, protogel) with 5 mL of 10x Tris-Boric acid buffer (890 mM each, pH 8.3 or pH 7.05 adjusted with HCl) and 20 mL of Milli-Q water. To this 400 µL of a 10% w/v ammonium persulfate/water solution and 40 µL of TEMED were added to initialise polymerisation. This is then immediately poured between 2 glass plates and a comb inserted at the top this is then left to set for 1 hr. Samples were made up to 10 µl containing 0.4 µM per strand, of each strand (S1*, S2 and S3 unless stated otherwise), 1xTBN buffer (89 mM Tris, 89 mM Boric acid, 10 mM NaCl) and the indicated ratio of Complex or competitor. DNA, water, and buffer were mixed in solution before addition of the stated ratios of complex and competitor. Samples were then incubated at 37 degrees Celsius for 1 hr. 5 µl of 30% w/v glycerol was then added to each sample and the sample was then centrifuged, mixed, and pipetted into the wells on the gel. The gel was run at 140 V for 2 hours in 1xTB buffer. The gel was then removed from the plates and placed into a phosphor imaging box with a screen and left for 2 hours, the screen was then removed and imaged on a BIO-RAD FarosFX Plus molecular imager.

Fluorescent PAGE competition experiments: PAGE Polyacrylamide gels were prepared by mixing 25 ml of 29:1 acrylamide/bis-acrylamide (National Diagnostic, protogel) with 5 mL of 10x Tris-Boric acid buffer (890 mM each, pH 8.3) and 20 mL of Milli-Q water. To this 400 µL of a 10% w/v ammonium persulfate/water solution and 40 µL of TEMED were added to initialise polymerisation. This is then immediately poured between 2 glass plates and a comb inserted at the top this is then left to set for 1 hr. Samples were prepared up to 30 uL with 1uM of S1-FAM, S2, and S3, and either 0, 0.5, 1, 2, or 4 equivalents of the competition strands and finally 1 equivalent of either Au pillarplex or Ni cylinder in 1xTBN (89 mM Tris, 89 mM Boric acid, 10 mM NaCl, at pH 8.3). Samples were incubated at room temperature for 1 hr before 7.5 uL of 50% glycerol solution was added to each solution. Sample aliquots of 15 uL were then loaded into the appropriate wells in the gel which was run for 1 hr at 140V in 1xTB buffer. The gel was imaged on an AlphaImagerTM Gel Imaging System From Alpha Innotech (the gel was subsequently stained SYBRTM Gold Nucleic Acid Gel Stain (Thermofisher scientific) in 1xTB buffer for 45 minutes and imaged again to confirm the presence of the expected structures). ImageJ was used to quantify the intensity of the 3WJ-FAM bands formed. The band intensities of 3WJ-FAM formed with Ni cylinder and competitors were compared to the intensity of the 3WJ-FAM band formed with Ni cylinder and No competitors to give a relative intensity. The

same process was carried out with lanes containing Au pillarplex. All experiments were undertaken as at least triplicate repeats.

Gel staining and quantification in (non-competition) fluorescent PAGE gels

The gels presented in the main manuscript are stained with SYBR gold. SYBR gold staining is widely used in nucleic acid gel electrophoresis and an advantage is that it stains all the different nucleic acid structures present. However SYBR does not stain all structures uniformly/equally; as such trends within a band can be discerned and potentially quantified, but relative quantification between different bands (structures) is not appropriate and can be misleading. Further complexity arises in this study because transition metal complexes are quenchers of organic fluorophores (through a variety of mechanisms including electron transfer). Thus it is not possible to directly compare band intensity across different metallo-supramolecular complexes (e.g. between the bands of Au pillarplex and Ni cylinder).

Pre-labelling the end of one strand with the FAM fluorophore (carboxyfluorescein) is a popular alternative approach to SYBR labelling, but also suffers from the issue of metal complex emission quenching. It also introduces a polyaromatic surface to which the complexes could bind.

Figure S21 compares these different approaches focusing on the gel in main manuscript Figure 3. Fig S21a compares the SYBR stained gel (from Fig.3) with the corresponding FAM-labelled gel and then with that FAM-labelled gel subsequently SYBR stained and re-imaged. The FAM-labelled gel and SYBR stained FAM-labelled gels have been quantified using Image-J (Schneider, C. A., Rasband, W. S., & Eliceiri, K. W. (2012). NIH Image to ImageJ: 25 years of image analysis. *Nature Methods*, *9*(7), 671–675. doi:10.1038/nmeth.2089) and the data are presented in Fig S21b and S21c.

Notable features are:

(i) The FAM-labelled gel indicates that Au pillarplex and Ni cylinder stabilise the 3WJ formation to a similar extent, but when the same gel is stained with SYBR and imaged, the Au pillarplex gives a stronger 3WJ band than the Ni cylinder, demonstrating that Au pillarplex and the Ni cylinder in complex with the 3WJ affect the signal of these two fluorophores (SYBR, FAM) to different extents (Fig. S21d).

(ii) At high loading (4:1) the Ni cylinder reduces the magnitude of the FAM fluorescence in the 3WJ band (middle gel lane 18 c.f. lanes 14-17). This may reflect additional Ni cylinders binding at the ends of the 3WJ DNA branches (as seen in the crystal structures in manuscript refs 28-30) and giving further quenching of the FAM. Alternatively the Ni cylinder (which absorbs at 305nm – Fig. S19b) may absorb light that would otherwise have excited the FAM.

(iii) The FAM fluorophore does bring additional complications with Au pillarplex binding to ssDNA when FAM is present causing appearance of a new band. This is not seen for Ni cylinder.

A radio-labelled gel (at 0.4 uM strand concentration) of 3WJ binding (Figs S21e,f), indicates that Au pillarplex and Fe cylinder at a 1:1 ratio induce the 3WJ formation to a similar extent and that the 3WJ is the dominant species. This confirms that the Au pillarplex and Ni cylinder are quenching the FAM fluorophore and doing so to a similar extent, such that the ssDNA band is overemphasised in the FAM fluorescence experiment. Since the Ni cylinder absorbs at 305nm but Au pillarplex does not,

quenching (rather than competitive light absorption) must be part of the mechanism of reduced FAM fluorescence.

In the context of the FAM competition assay (main manuscript Figure 10; SI Fig. S20) the issue of interference by metallo-drug fluorescence quenching is avoided since the same 3WJ band is monitored and metal complex ratios kept constant. Since the extent of induction of the FAM-3WJ band is similar for both Au pillarplex and Ni cylinder at 1:1, a qualitative comparison of the two competition graphs is possible.

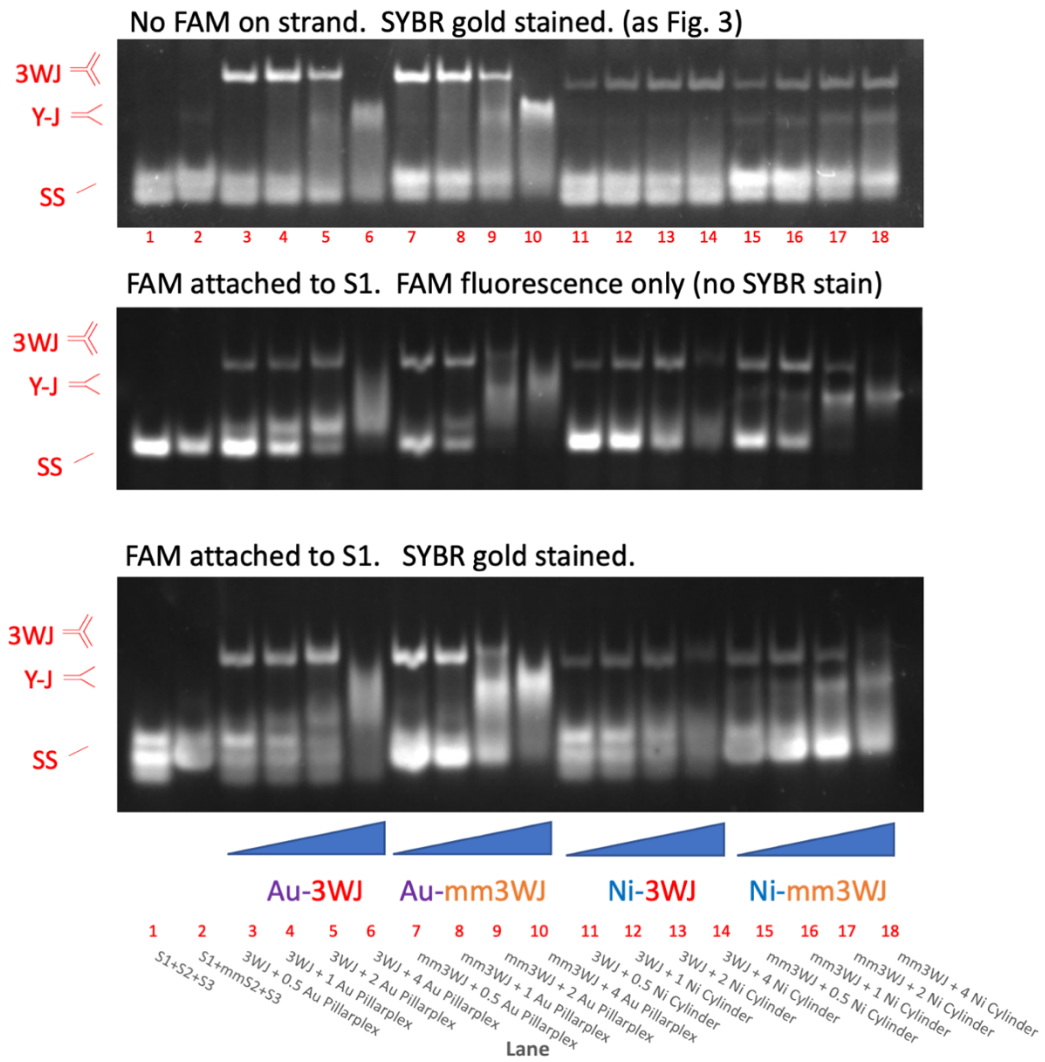

Figure S21a: Fluorescence gels with SYBR staining and FAM labelling

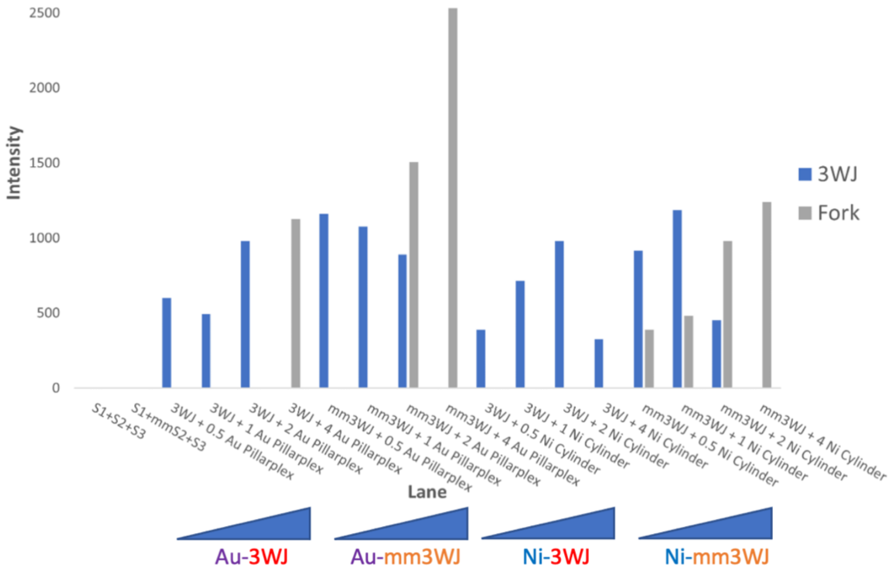

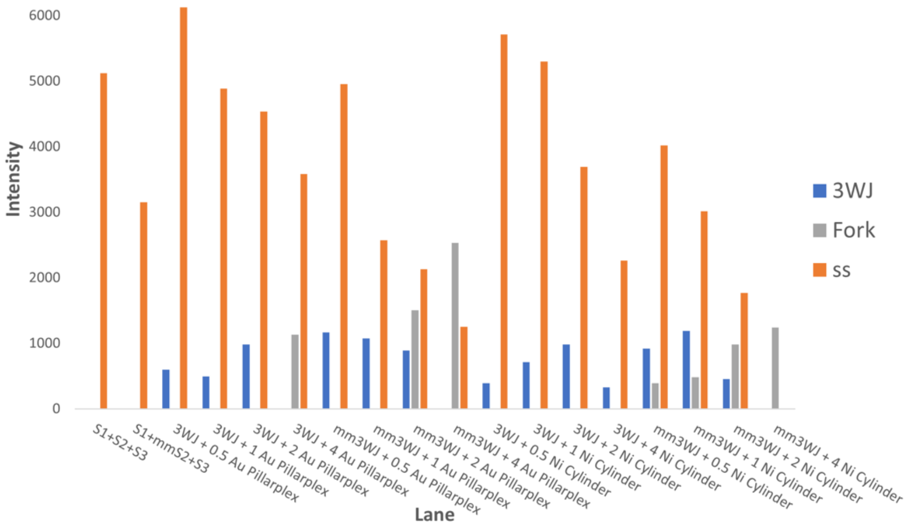

Figure S21b: Fluorescence emission from the bands in the FAM-labelled (no SYBR staining) gel. Top: 3WJ and Y-fork only. Bottom: also including ssDNA. Data are presented 'raw' rather than normalised because of the comparatively large response of the stains to the ssDNA band.

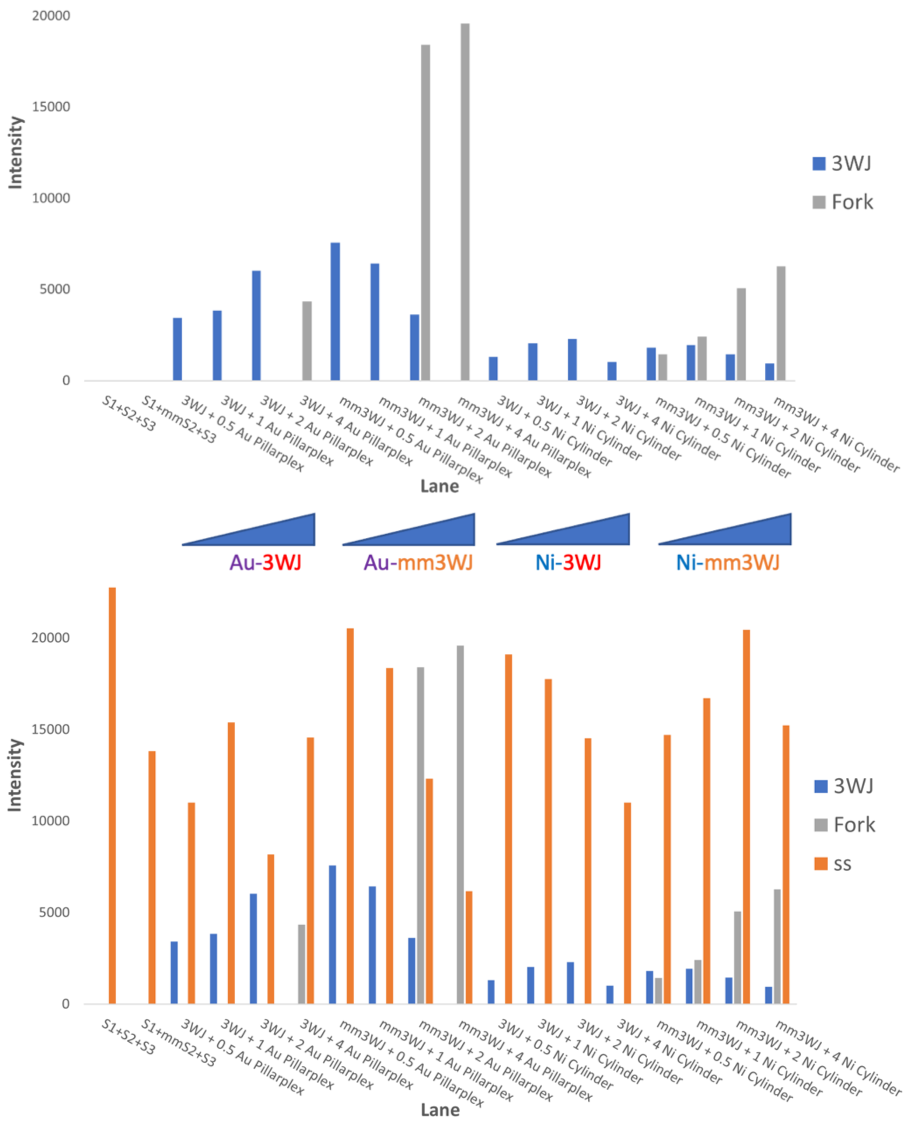

Figure S21c: Fluorescence emission from the bands in the SYBR stained, FAM-labelled gel. Top: 3WJ and Y-fork only. Bottom: also including ssDNA. Data are presented 'raw' rather than normalised because of the comparatively large response of the stains to the ssDNA band.

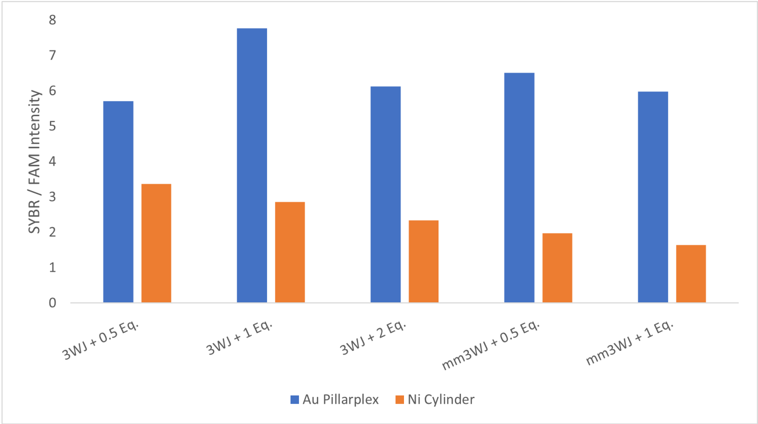

Figure S21d: Ratio of SYBR staining intensity to FAM intensity in the 3WJ bands in the FAM-labelled gel showing the difference between Au pillarplex and Ni cylinder.

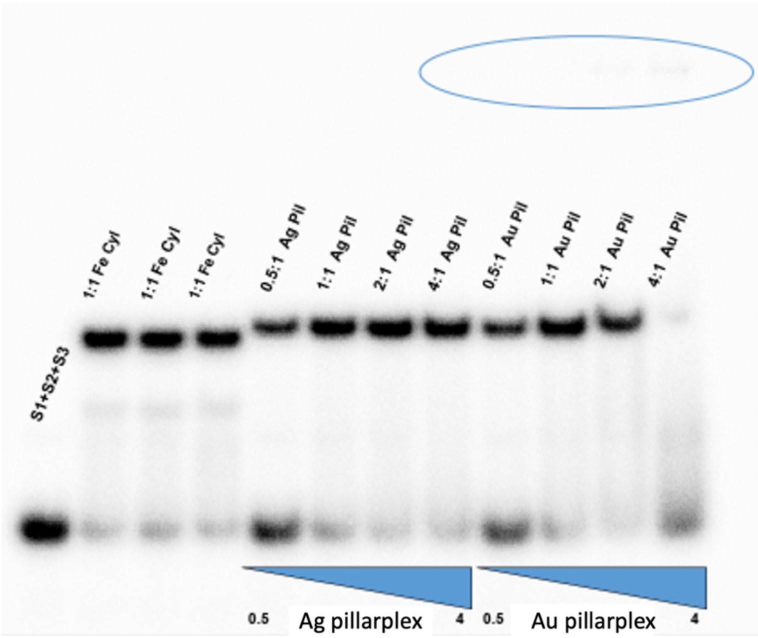

Figure 21e: ^{32}P radio-labelled 3WJ gel (0.4 uM strands) with iron(II) cylinder, Ag pillarplex and Au pillarplex. Blue circle highlights trace slow running species (likely some small amounts of well precipitation).

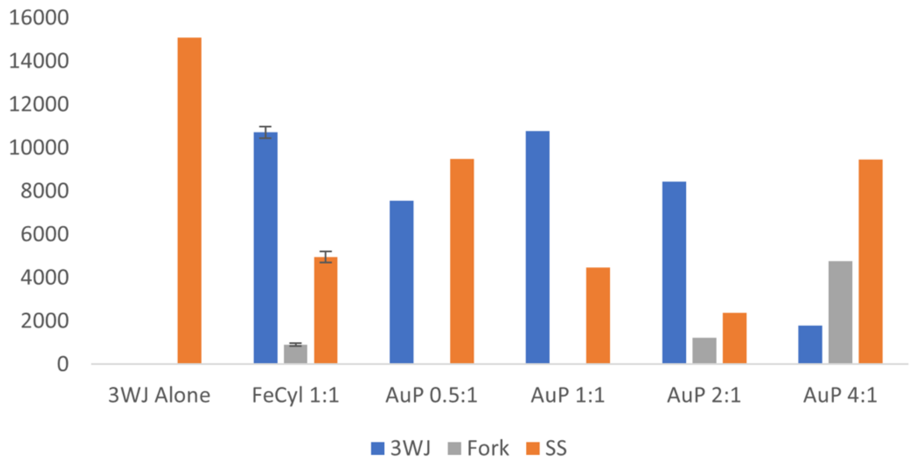

Figure 21f: Band intensities from the Figure 21c radio-labelled 3WJ gel. Smearing of the Y-fork band for Au pillarplex hindered accurate quantification of that band, (underestimated) in the 2:1 and 4:1 ratios.

Agarose gel electrophoresis: Agarose gels were prepared by mixing 4 g of agarose (UltraPure™ Agarose, thermofisher scientific) with 400 ml of Milli-Q water (18.2 MΩ) containing 1x Tris-Boric acid buffer (890 mM each, pH 8.3). This was microwaved till all the solid had dissolved and cast into the agarose gel tray with a 15-lane comb. DNA/complex samples were prepared in 1xTB buffer with 30 µM DNA in base pairs and the labelled ratio of complex to this. Samples were 20 µl in total, to this, 10 µl of 30% w/v glycerol solution was added for sample loading into the gel. A DNA ladder was used in the left and right most lanes with 10, 4 and 0.5 kDa mass, top to bottom. The Gel was run at 140 V for 2 hours in 1xTB buffer. The gel was then removed from

the plates and stained using SYBR™ Gold Nucleic Acid Gel Stain (Thermofisher scientifc) in 1xTB buffer for 45 minutes before imaging on a bio-rad ChemiDoc fluorescent imager with 305 nm excitation.

pBR322 plasmid linearization preparation: pBR322 plasmid DNA was purchased from MERCK, linearisation was achieved using the pst1-HF restriction endonuclease (new England biolabs). 20 µl of 0.5 µg/µl pBR-322 DNA solution was incubated with 10 µl of pst1-HF (20,000 units/ml), 50 µl of 10x cutsmart buffer and 420 µl Milli-Q water (18.2 MΩ) were incubated together at 37 ºC for 1 hr. This reaction mixture was then purified using QAIquick PCR purification columns and eluted with 50 µl Milli-Q water (18.2 MΩ). Concentrations in DNA base pairs was determined using a multichannel nanodrop 8000 spectrophotometer by using the absorbance at 260 nm and ε260 = 13,200 mol^{-1} dm^3 cm^{-1} using the beer lambert law. Confirmation of the linearization was confirmed using agarose gel electrophoresis by observing the characteristic band shift and by comparison to the 4 kDa band in the DNA ladder.

Antiproliferative assays Human lung adenocarcinoma (A549) cells were obtained from American Type Culture Collection (ATCC). The cells were cultured in Dulbecco's Modified Eagle Medium (DMEM, 4.5 g/L glucose Corning), supplemented with 10% fetal bovine serum (FBS, One shot, Gibco) and 1% penicillin/streptomycin (Gibco) and incubated in a humidified atmosphere of 5% CO_2 at 37 °C. To evaluate the antiproliferative effects of the compounds, cells were seeded in 96-well plates (Costar, Corning) at a concentration of 8000 cells/well and grown for 24 h in 200 µL culture medium. Solutions of the pillarplexes with the required concentration (1 to 100 µM) were prepared by diluting a freshly prepared stock solution (10^{-2} M in MilliQ) of the corresponding compound in aqueous DMEM medium, accordingly. After 24 h of incubation, 200 µL of the compounds' dilutions in DMEM medium were added to each well and the cells were incubated for 24 h or 72 h, under standard culture conditions. Afterwards, the medium was replaced with a 3-(4,5-dimethyl-2-thiazoyl)-2,5-diphenyltetrazolium bromide (MTT, Fluorochem) solution in 10x PBS (Corning) at a final concentration of 0.5 mg/mL and incubated for 3-4 h. Following incubation, the MTT solution was aspirated from the wells and the purple formazan crystals were dissolved in DMSO. Absorbance at 550 nm was determined in quadruplicates for each condition, using a multi-well plate spectrophotometer (Victor X5, Perkin Elmer). Cell viability was determined as the ratio of absorbance between treated and untreated cells (untreated controls). The EC_{50} value was calculated as the concentration showing a 50% reduction in cell viability, when compared to untreated controls, using a nonlinear fit of cell viability vs dose with GraphPad Prism 7 software. Data is represented as mean ± SEM of at least three independent experiments.

Cell uptake ICP-MS analysis For metal uptake/accumulation studies, cells were seeded in T25 flasks (Corning), grown to approximately 50% confluency and incubated with the corresponding metallodrug (dissolved directly in the incubation medium) at 6 µM for 24 h. At the end of the incubation period, cells were washed three times with ice cold PBS (containing 10 mM phosphate) before being detached using an enzyme free dissociation solution (Millipore, UK). The cells were counted after trypan blue staining in a haemocytometer, and the final suspension kept for analysis on the whole cell metal content. For whole cell extract analysis, cells were pelleted for 5 min at 700 x g and 4 °C and washed once with ice cold PBS. Cell lysis was achieved using a freeze-thaw technique suitable for cell uptake studies. Cold RIPA buffer (ThermoFisher Scientific) at a concentration of 1 mL per 5 million cells was added, followed by 30 sec of sonification with 50% pulse. After 15 min incubation

on ice, the mixture was centrifuged at 14.000 xg at 4 °C for 15 minutes to pellet debris. Supernatant was transferred to a new Eppendorf and both were frozen at -80 °C. All samples were analyzed for their protein content prior to ICP-MS determination. For cell fractionation studies, the cell suspension following compound's treatment and PBS washing steps was divided into two equal aliquots. One aliquot was pelleted and kept for analysis on the whole cell, while the second aliquot was treated for cell fractionation as described below. For cell fractionation, the Nuclear/Cytosol Extraction Kit (Biovision Inc.) was used. All samples were digested in concentrated nitric acid for 3 h and filled to a total volume of 8 ml with a mixture HCl/water. Indium was added as an internal standard at a concentration of 0.5 ppb. The metal content was quantitated by inductively coupled plasma mass spectrometry (ICP-MS), using an ICP-MS Agilent 7900 (Agilent Technologies, Cheshire, UK) instrument, equipped with an internal autosampler and a nebulizer at a sample uptake rate of 0.25 ml/min. The instrument was calibrated on a daily basis. ICP-MS parameters were: RF power 1560 W, dwell time 0.3 s, replicates 10, monitored isotopes ^{197}Au and ^{109}Ag. Au and Ag standards for ICP-MS measurements were derived from PlasmaCAL standards (SCP Science, Quebec, Canada). The Agilent MassHunter software package was used for data processing. The obtained results are the average ± SE of at least three experiments.

Compounds: Gold and silver Pillarplexes and the nickel and iron Cylinders $[M_2L_3][Cl]_4$ were prepared as previously described in:

Altmann, P.J.; Pöthig, A. Pillarplexes: A Metal–Organic Class of Supramolecular Hosts. *J. Am. Chem. Soc.* **2016**, *138* (40), 13171–13174.

Hannon, M.J.; Painting, C.L.; Jackson, A.; Hamblin, J.; Errington, W. An inexpensive approach to supramolecular architecture. *Chem. Commun.* **1997**, 1807–1808.

Kerckhoffs, J.M.C.A.; Peberdy, J.C.; Meistermann, I.; Childs, LJ.; Isaac, C.J.; Pearmund, C.R.; Reudegger, V.; Khalid, S.; Alcock, N.W.; Hannon M.J.; Rodger, A. Enantiomeric resolution of supramolecular helicates with different surface topographies. *Dalton Trans.*, **2007**, 734-742

Contributions

MJH and AP conceived, supervised and directed the project. JSC undertook gel and spectroscopic experiments, and designed experiments with MJH. HDW and SJD undertook gel and UV melting experiments and CC undertook gel experiments. DFB supervised radio-labelled gels. LM designed and with SJD undertook the MD simulations. RKOS, SJ and RE investigated oligonucleotide NMR and crystallization studies. AP, AAH, PJA and SG prepared the pillarplexes and ligand, and JSC prepared the cylinders. AC and BA contributed the antiproliferative activity and cellular accumulation studies. MJH and JSC drafted the manuscript which all authors commented on.

Molecular Dynamics Simulations

Parameterisation of the gold pillarplex and iron(II) cylinder. The iron(II) cylinder was parameterised using the MCPB.py pipeline as detailed previously [5]. Atomistic parameterisation of the organometallic gold pillarplex within the MCPB.py pipeline [1], brought additional challenges with the coordinating carbene carbons initially not properly described as part of their aromatic rings. To address this and retain aromaticity of these carbons, the appropriate atom type from the general AMBER force field (GAFF) was chosen (carbon in a 5 membered ring adjacent to nitrogen). Parameters for the coordination bond were then calculated using the MCPB.py pipeline with Gaussian09 at the CAM-B3LYP/DEF2-SVP level of theory to include dispersion, with ECP for Au. The resultant parameters and topology files were converted to GROMACS format using ParmEd (https://github.com/ParmEd/ParmEd).

Parameterisation of DNA. The B-DNA structure file was generated with random sequence using NAB (nucleic acid builder) in AmberTools [3]. The AAA-3WJ structure was taken from the 3I1D PDB crystal structure after removing the helicate. The AGG-3WJ was generated from the AAA-3WJ by mutating two of the adenines at the junction point into guanines, and the corresponding thymines into cytosines, using the mutagenesis tool in PyMOL [4]. The larger 3WJ was taken from PDB 1F44. After removing the solvent molecules and the proteins, the gaps in the backbones were joined up using Avogadro to create three continuous DNA strands. The structure was then energy minimised in GROMACS [10]. To improve computational expense, each strand was shortened to 14 bases. The 4WJ structure was taken from the 1XNS PDB crystal structure after removal of the peptides and water molecules, and each arm of the DNA structure was shortened by 9 base pairs to reduce the computational cost of modelling the 4WJ. [5] All DNA was parameterised using the AMBER forcefield parmbsc1. [6]

Molecular Docking. Autodock Vina (version 1.1.2) [7-8] was used on Castles red VM [9] to place the pillarplex on the 3WJ. As Autodock Vina has no parameters for Au atoms, they were replaced with Zn for the docking calculation. The resulting structures were only used as a starting point for MD simulations and are not physically relevant, as (a) the 3WJ without a ligand is not a stable structure and (b) the Au parameters for docking were oversimplified.

Molecular Dynamics Simulations. In simulations with the 4WJ, the pillarplex was placed directly at/inside the open cavity of the structure. (When placed further away the cavity closed before the pillarplex could enter and did not reopen in microsecond timeframes). In all simulations of the B-DNA and 3WJ, DNA was placed with the pillarplex (or cylinder) in a dodecahedral box with periodic boundary conditions. In all simulations of the 4WJ, DNA was placed with the pillarplex in a cubic box with periodic boundary conditions. All systems were solvated in water using the TIP3P model and neutralised with Na+ ions. Additional Na+ and Cl- ions were added to reach a NaCl concentration of 50mM. Using GROMACS software [10] initial minimisation was carried to at least 500 kJ/mol/nm or 50000 steps followed by heating and NVT equilibration for 1000ps using V-rescale modified Berendsen thermostat, coupling the cylinder with the DNA at 310K. All simulations use 2 fs time step and Parrinello-Rahman pressure coupling and PME electrostatics at 1.0nm cut-off. All simulations were run on the BlueBEAR cluster at U. Birmingham. After the

simulations had finished, the trajectories were processed in GROMACS to remove periodic boundary conditions, translations and rotations, and visualised in PyMOL. Representative RMSD plots are included in Figure S22.

[1] P. Li, K.M. Merz, Jr. *J. Chem. Inf. Model.* 2016, 56, 599–604
[2] L. Melidis, I. B. Styles, M. J. Hannon, Chem. Sci., 2021,12, 7174-7184; L. Melidis, H. J. Hill, N. J. Coltman, S. P. Davies, K. Winczura, T. Chauhan, J. S. Craig, A. Garai, C. A. J. Hooper, R. T. Egan, J. A. McKeating, N. J. Hodges, Z. Stamataki, P. Grzechnik, M. J. Hannon, *Angew. Chem. Int. Ed.* **2021**, *60*, 18144.
[3] D.A. Case, K. Belfon, I.Y. Ben-Shalom, S.R. Brozell, D.S. Cerutti, T.E. Cheatham, III, V.W.D. Cruzeiro, T.A. Darden, R.E. Duke, G. Giambasu, M.K. Gilson, H. Gohlke, A.W. Goetz, R Harris, S. Izadi, S.A. Izmailov, K. Kasavajhala, A. Kovalenko, R. Krasny, T. Kurtzman, T.S. Lee, S. LeGrand, P. Li, C. Lin, J. Liu, T. Luchko, R. Luo, V. Man, K.M. Merz, Y. Miao, O. Mikhailovskii, G. Monard, H. Nguyen, A. Onufriev, F. Pan, S. Pantano, R. Qi, D.R. Roe, A. Roitberg, C. Sagui, S. Schott-Verdugo, J. Shen, C.L. Simmerling, N.R. Skrynnikov, J. Smith, J. Swails, R.C. Walker, J. Wang, L. Wilson, R.M. Wolf, X. Wu, Y. Xiong, Y. Xue, D.M. York and P.A. Kollman (2020), AMBER 2020, University of California, San Francisco.
[4] The PyMOL Molecular Graphics System, Version 1.3, Schrödinger, LLC.
[5] K. Ghosh, C.K. Lau, F. Guo, A.M. Segall, G.D. Van Duyne, *J. Biol. Chem.,* 2005, 280, 8290-8299
[6] I. Ivani, P.D Dans, A. Noy, A. Pérez, I. Faustino, A. Hospital, J. Walther, P. Andrio, R. Goñi, A. Balaceanu, G. Portella, F. Battistini, J.L. Gelpí, C. González, M. Vendruscolo, C.A Laughton, S.A Harris, D.A Case M. Orozco *Nat Methods* 2016, 13, 55–58
[7] J. Eberhardt, D. Santos-Martins, A. F. Tillack, and S. Forli. AutoDock Vina 1.2.0: New Docking Methods, Expanded Force Field, and Python Bindings. J*. Chem. Inf. Model.* 2021, 61, 8, 3891–3898.
[8] O. Trott, A. J. Olson, AutoDock Vina: improving the speed and accuracy of docking with a new scoring function, efficient optimization and multithreading, J. Comp. Chem., 2010, 31, 455-461
[9] Thompson, S.J.; Thompson, S.E.M.; Cazier, J.-B. Castles (compute and Storage for the Life Sciences): A Collection of Compute and Storage Resources for Supporting Research at the University of Birmingham. *Zenodo* June 20, 2019 https://doi.org/10.5281/zenodo.3250616.
[10] M.J. Abraham, T. Murtol, R. Schulz, S. Páll, J.C. Smith, B. Hess, E. Lindahl, *SoftwareX,* **2015,** *1–2, ,* 19-25*, https://doi.org/10.1016/j.softx.2015.06.001*
[11] M.D. Hanwell, D.E. Curtis, D.C. Lonie, T. Vandermeersch, E. Zurek, G.R. Hutchison; Avogadro: An advanced semantic chemical editor, visualization, and analysis platform. *J. Cheminformatics*, 2012, 4, 17.

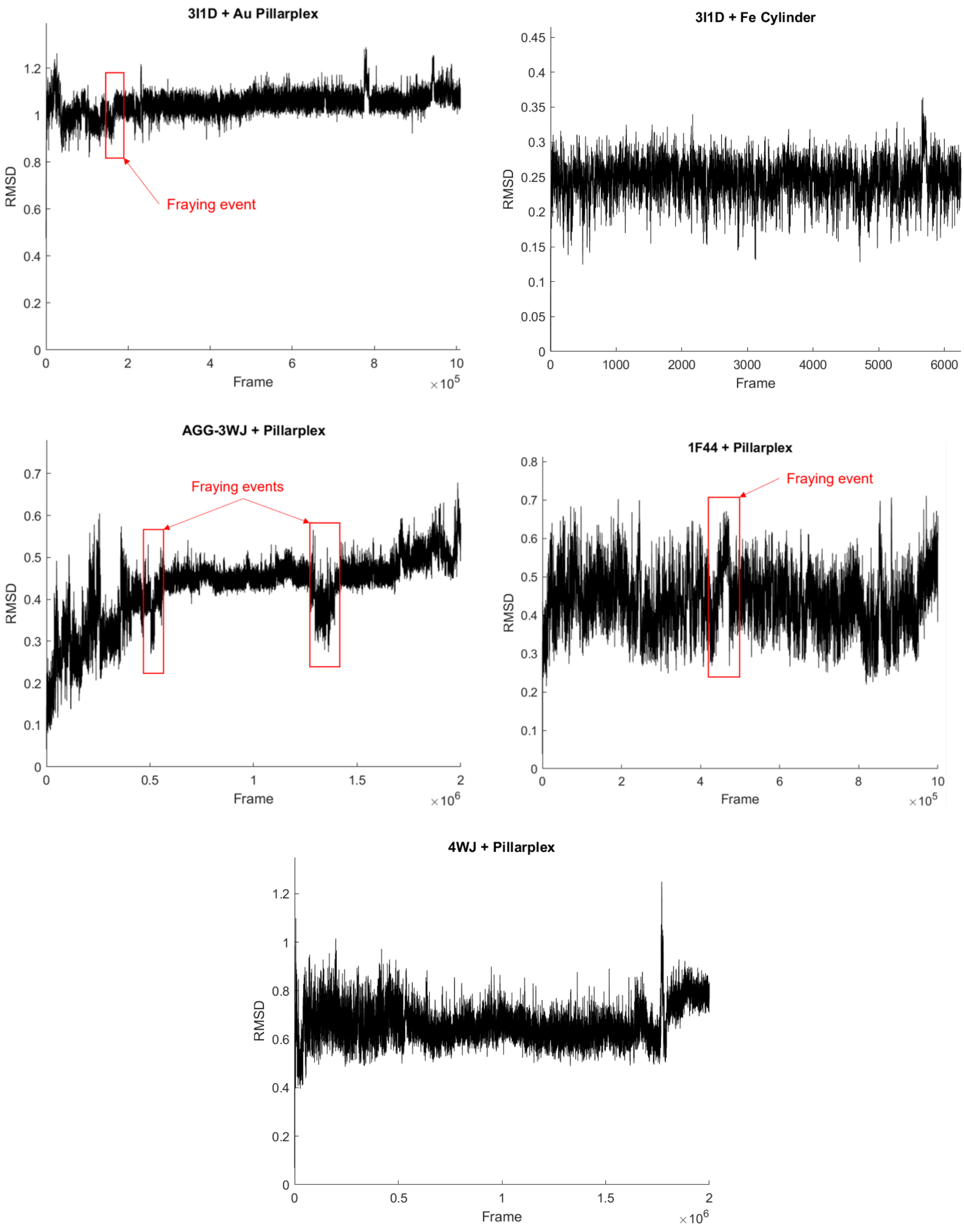

Figure S22: Representative RMSD plots for the simulations of 3WJ (from pdb 3I1D), AGG-3WJ, longer 3WJ (derived from pdb 1F44) and 4WJ.
